# Transcriptome analysis reveals regulatory framework for salt and osmotic tolerance in a succulent xerophyte

**DOI:** 10.1186/s12870-019-1686-1

**Published:** 2019-02-28

**Authors:** Hongju Yin, Mengzhan Li, Dingding Li, Sardar-Ali Khan, Shelley R. Hepworth, Suo-Min Wang

**Affiliations:** 1 State Key Laboratory of Grassland Agro-ecosystems; Key Laboratory of Grassland Livestock Industry Innovation, Ministry of Agriculture and Rural Affairs; College of Pastoral Agriculture Science and Technology, Lanzhou University, Lanzhou, 730020 People’s Republic of China; 2 Department of Biology, Institute of Biochemistry, Carleton University, Ottawa, ON Canada

**Keywords:** RNA-sequencing, Osmotic stress, Salt, Protein kinases, Transcription factors, Proteolysis

## Abstract

**Background:**

*Zygophyllum xanthoxylum* is a succulent xerophyte with remarkable tolerance to diverse abiotic stresses. Previous studies have revealed important physiological mechanisms and identified functional genes associated with stress tolerance. However, knowledge of the regulatory genes conferring stress tolerance in this species is poorly understood.

**Results:**

Here, we present a comprehensive analysis of regulatory genes based on the transcriptome of *Z. xanthoxylum* roots exposed to osmotic stress and salt treatments. Significant changes were observed in transcripts related to known and obscure stress-related hormone signaling pathways, in particular abscisic acid and auxin. Significant changes were also found among key classes of early response regulatory genes encoding protein kinases, transcription factors, and ubiquitin-mediated proteolysis machinery. Network analysis shows a highly integrated matrix formed by these conserved and novel gene products associated with osmotic stress and salt in *Z. xanthoxylum*. Among them, two previously uncharacterized NAC (NAM/ATAF/CUC) transcription factor genes, *ZxNAC083* (Unigene16368_All) and *ZxNAC035* (CL6534.Contig1_All), conferred tolerance to salt and drought stress when constitutively overexpressed in Arabidopsis plants.

**Conclusions:**

This study provides a unique framework for understanding osmotic stress and salt adaptation in *Z. xanthoxylum* including novel gene targets for engineering stress tolerance in susceptible crop species.

**Electronic supplementary material:**

The online version of this article (10.1186/s12870-019-1686-1) contains supplementary material, which is available to authorized users.

## Background

Drought and salinity are two major environmental stressors that impact crop yields worldwide [1]. Salinity threatens approximately one-fifth of cultivated lands globally. Meanwhile, up to one third of land surfaces are exposed to drought [2, 3]. Many experts agree that using molecular genetics to breed crops with higher yields and improved tolerance to abiotic stresses is an effective strategy in safeguarding food supplies. Yet, this task remains one of the greatest challenges faced by modern agriculture [4]. Deciphering key genes and regulatory mechanisms in salt and drought tolerance and adaptation is a key step in engineering stress-tolerant crop plants [5, 6].

Mechanisms of abiotic stress tolerance have mainly been studied in model plants. High throughput sequencing and functional genomics tools in model plants have yielded numerous abiotic stress tolerance genes grouped into two major classes: functional genes or regulatory genes [7]. Functional genes include important enzymes and metabolic proteins including detoxification enzymes, water channels, ion transporters, heat shock proteins and late embryogenesis abundant proteins, which directly function to protect cells from stress. Regulatory genes include important signaling and regulatory proteins that modulate protein activity during stress exposure including hormone and stress signaling pathway components, transcription factors, and ubiquitin-mediated proteolysis machinery. A large number of studies show that scope for enhancing stress tolerance in plants by altering single functional genes is limited, due to the complexity of stress responses [8, 9]. Thus, alteration of key regulatory genes is desirable because this can mimic or enhance stress signals by regulating a large spectrum of downstream stress-responsive genes in conferring tolerance [10, 11]. Knowledge of regulatory genes is therefore important from an genetic engineering perspective in plants [11, 12].

Despite vast knowledge derived from *Arabidopsis thaliana* (Arabidopsis) and *Oryza sativa* (rice) model plants, a low capacity for stress tolerance limits their usefulness as discovery tools. By contrast, xerophyte and halophyte species, widely distributed in arid and saline regions, have evolved multiple protective mechanisms that allow them to grow successfully under hostile conditions [6, 13, 14]. A detailed understanding of salt and drought protective mechanisms in naturally tolerant species, and the identification of key regulatory genes, is a promising new strategy for breeding salt and drought tolerant crops [13, 15].

*Zygophyllum xanthoxylum* is a succulent xerophyte with a highly developed root system and strong stress tolerance. The natural range of *Z. xanthoxylum* includes arid and semiarid lands in northwestern China and Mongolia [16]. This shrub is widely planted in China for protecting fragile desert ecosystems and improving vegetation coverage [17]. Previous investigations in *Z. xanthoxylum* have focused on growth properties, nutritive characteristics, and transpiration resulting in the characterization of several drought and salt stress response functional genes [17–22]. Previously, we ge nerated transcriptome datasets of roots and leaves of *Z. xanthoxylum* to identify differentially expressed genes (DEGs) under osmotic stress and salt treatments [20]. Attention was focused on several important classes of functional genes traditionally associated with drought and salt stress responses, including ion transporters, reactive oxygen species (ROS) scavenging systems, and photosynthesis [20].

**Fig. 1:**
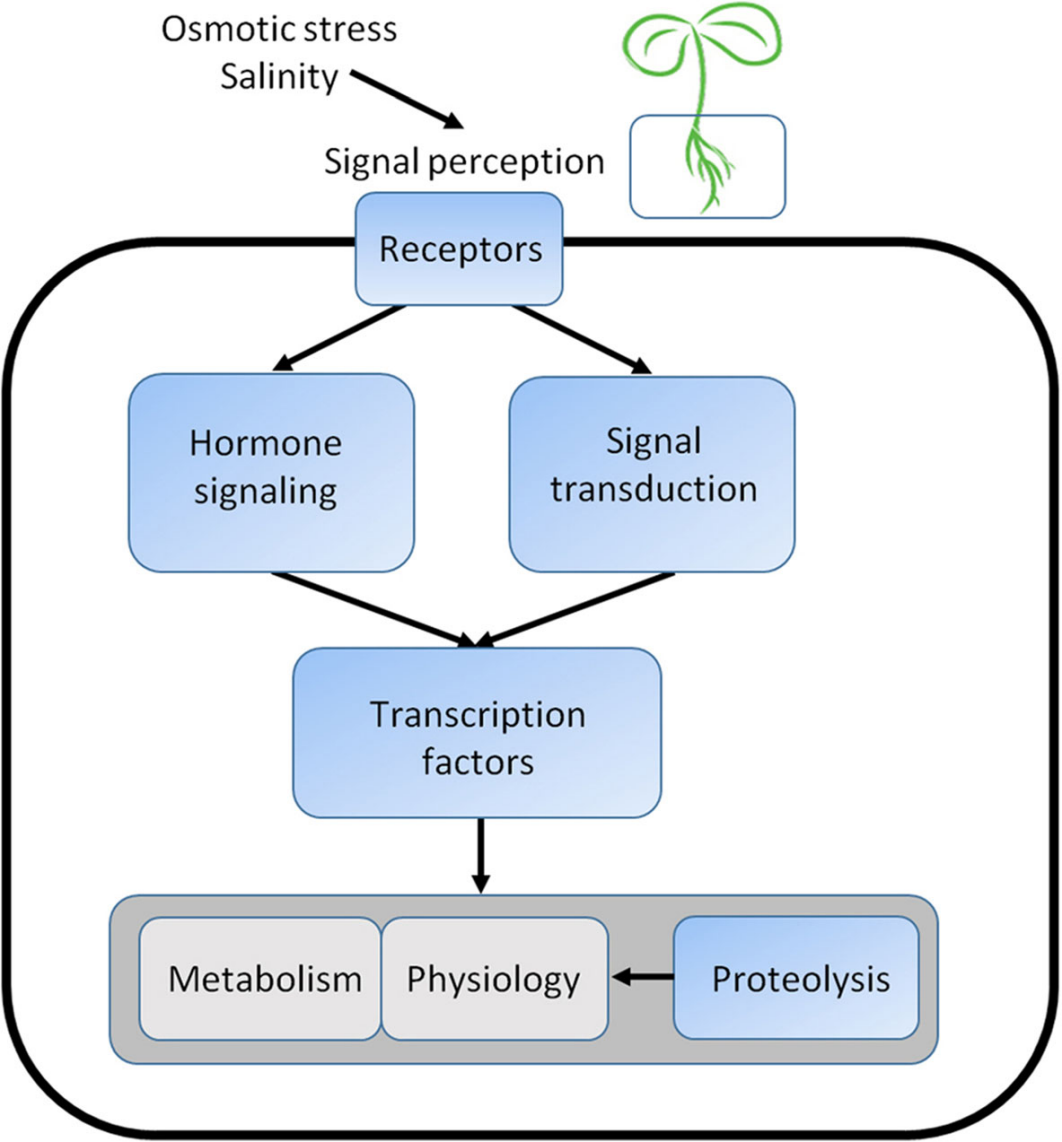
An overview of relationships between the regulatory gene groups studied in this work and their downstream functional genes. The 6 h and 24 h DEG transcriptomes of *Z. xanthoxylum* roots under osmotic stress and salt treatment were analyzed. Regulatory gene categories, blue boxes. Functional gene categories, gray boxes

Here, we further analyzed the transcriptome and digital gene expression profiling data of *Z. xanthoxylum* roots under osmotic stress and salt treatments to identify potential upstream regulators of these functional genes (Fig. 1). Our transcriptome analysis focused on signaling pathways important for stress tolerance, transcription factors important for signal output, and ubiquitin proteasome system enzymes important for protein turnover (Fig. 1). To further analyze this transcriptome, DEGs in *Z. xanthoxylum* roots were matched to putative orthologs in Arabidopsis, which allowed us to perform in silico functional inference, including gene network analysis for protein function, protein subcellular localization, and gene co-expression. Finally, candidate genes were selected and functionally characterized. Among these, *Z. xanthoxylum* unigenes orthologous to Arabidopsis NAC transcription factor genes *NAC083* and *NAC035* conferred drought and salt tolerance when constitutively overexpressed in Arabidopsis plants, confirming relevance of the dataset for crop engineering.

## Results and discussion

### Transcriptome profile of roots during osmotic stress and salt treatments

To learn more about how *Z. xanthoxylum* adapts to drought and saline environments, we further analyzed the transcriptome data of seedling roots exposed to osmotic stress and salt treatments for 6 h and 24 h [20]. Roots were studied because they are the first organs to be exposed to osmotic and salt stress conditions [23]. 6063 and 6258 DEGs were identified in *Z. xanthoxylum* roots exposed to osmotic stress or salt treatments for 6 h, respectively (Additional file 1: Figure S1a). In osmotic-stressed seedling roots, 4000 DEGs were up-regulated and 2063 DEGs were down-regulated. In salt-treated seedling roots, 4140 DEGs were up-regulated and 2118 DEGs were down-regulated (Additional file 1: Figure S1a). Venn-diagram analysis shows that a large number of 6 h DEGs overlap in expression, including 2780 up-regulated genes and 1402 down-regulated genes that are present in both osmotic stress and salt treatments, suggesting that signaling pathways controlling these responses in *Z. xanthoxylum* are interacting (Additional file 1: Figure S1a). By comparison, only 2708 and 1307 DEGs were identified in roots exposed to osmotic stress or salt treatments for 24 h, respectively (Additional file 1: Figure S1b). In osmotic-stressed seedling roots, 1723 DEGs were up-regulated and 985 DEGs were down-regulated. In salt-treated seedling roots, 657 DEGs were up-regulated and 750 DEGs were downregulated (Additional file 1: Figure S1b). Similarly, Venn-diagram analysis revealed that 329 up-regulated genes and 309 down-regulated genes overlapped in osmotic stress- and salt-treated seedling roots. Overall, the 6 h dataset was more complex than treatment for 24 h, reflecting that regulatory genes are generally early responders to environmental signals [7, 20]. We therefore selected the 6 h dataset as the focus of further analysis.

### Functional assignment of DEGs under osmotic stress and salt treatment

**Fig. 2:**
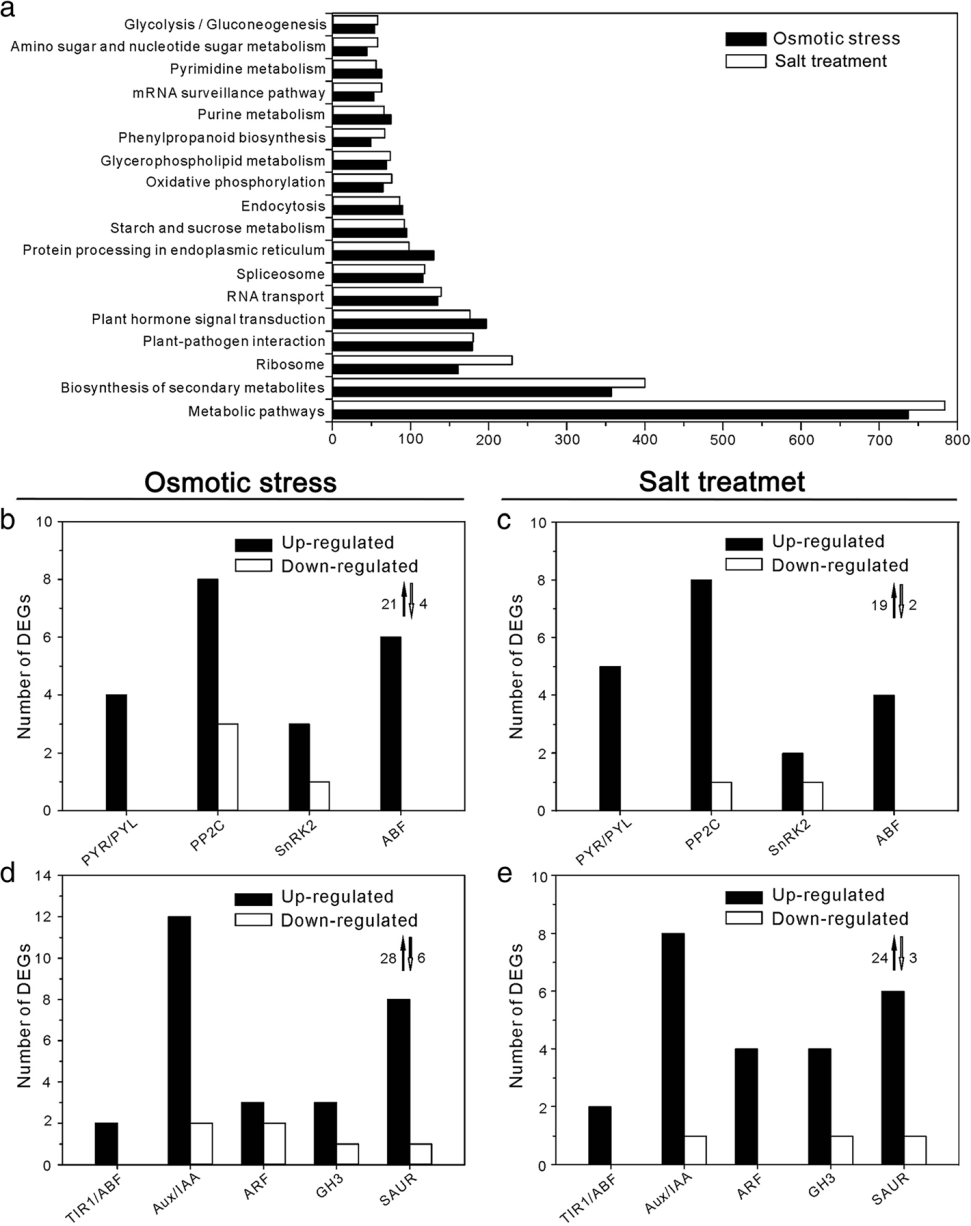
KEGG analysis of DEGs in *Z. xanthoxylum* roots. **a** Top 18 enriched functional categories and distribution of DEGs in (**b, c**) ABA and (**d, e**) auxin signaling pathways under (**b, d**) osmotic stress and (**c, e**) salt treatment. **a** X-axis indicates the number of DEGs in KEGG pathways, and Y-axis represents the specific category of DEGs in the pathway. Black bars, number of up-regulated genes. White bars, number of down-regulated genes. Black arrows, total number of up-regulated genes. White arrows, total number of down-regulated genes

To evaluate the biological pathways and molecular function of genes participating in osmotic stress and salt response, DEGs in 6 h-treated *Z. xanthoxylum* roots were functionally annotated by aligning their sequences to proteins in the KEGG database (see Methods). 3028 DEGs and 3167 DEGs were functionally annotated under osmotic stress and salt treatment, respectively. Eighteen functional pathways were identified as significantly enriched (*p* ≤ 0.05) during both treatments (Fig. 2a). In osmotic-stressed *Z. xanthoxylum*, the dominant pathways were “metabolic pathways” (737 DEGs), “biosynthesis of secondary metabolites” (357 DEGs), “plant hormone signal transduction” (197 DEGs) “plant-pathogen interaction” (179 DEGs), “ribosome” (161 DEGs), “RNA transport” (135 DEGs) and “spliceosome” (116 DEGs). In salt-treated *Z. xanthoxylum*, the dominant pathways were “metabolic pathways” (784 DEGs), “biosynthesis of secondary metabolites” (400 DEGs), “ribosome” (230 DEGs), “plant-pathogen interaction” (180 DEGs), “plant hormone signal transduction” (179 DEGs), “RNA transport” (139 DEGs) and “spliceosome” (118 DEGs) (Fig. 2a).

Among hormone signaling pathways, abscisic acid (ABA) signaling components were significantly enriched followed by auxin, ethylene, and cytokinin signaling pathway terms. ABA plays a well-established role in plant stress response signaling including salinity, drought, osmotic, and cold stresses [24]. During stress responses, cellular ABA levels increase via ABA biosynthesis. PYL/PYR/RCAR (PYRABACTIN RESISTANCE1/PYR1-LIKE/REGULATORY COMPONENTS OF ABA RECEPTORS) receptors bind to ABA and interact with PP2C (protein phosphatase 2C) negative regulators thereby releasing SnRKs (sucrose non-fermenting like kinases). Activated SnRKs phosphorylate downstream proteins, including bZIP (basic leucine zipper) transcription factors, thereby activating stress tolerance genes [24, 25]. DEGs encoding all of these core positive components of ABA signaling were upregulated in *Z. xanthoxylum* roots under osmotic stress and salt treatments (Fig. 2b and c, Additional file 2: Table S1). PP2C components showed the most change, with 8 up-regulated and 3 down-regulated genes under osmotic stress conditions and 8 up-regulated and 1 down-regulated gene under salt conditions (Fig. 2b and c, Additional file 2: Table S1). These data indicate that ABA signaling is an important factor in *Z. xanthoxylum* stress tolerance, similar to other plants. ABA signaling directly activates stress tolerance genes as well as initiates secondary mechanisms for stress tolerance [26]. Auxin is an important regulator of plant growth and development but its role in abiotic stress responses is poorly studied [27]. Interestingly, numerous auxin signaling components were differentially expressed in osmotic-stressed and salt-treated *Z. xanthoxylum* roots. Under osmotic stress, 28 auxin signaling pathway genes were upregulated, including 2 TIR1/AFB (Transport Inhibitor Response1/Auxin Signaling F-box) receptor genes, 12 AUX/IAA (Auxin/Indole-3-Acetic Acid) repressor genes, 3 ARF (Auxin Response Factor) transcription factor genes, 3 GH3 (Gretchen Hagen 3) and 8 SAUR (Small Auxin-Up RNA) auxin early-response genes (Fig. 2d and Additional file 2: Table S2). Under salt stress, 24 auxin signaling pathway genes were upregulated including 2 TIR/AFB genes, 8 AUX/IAA genes, 4 ARF genes, 4 GH3 genes, and 6 SAUR genes (Fig. 2e and Additional file 2: Table S2). This enrichment suggests that auxin signaling is an important element in *Z. xanthoxylum* stress tolerance. Unlike Arabidopsis, *Z. xanthoxylum* can effectively maintain growth and regulate its root architecture under osmotic stress and salinity [18–20]. In turn, auxin plays a well-characterized role in stimulating root growth [27]. Collectively, steep changes in ABA and auxin signaling genes at 6 h under osmotic-stress and salt treatments suggests an important interconnecting role for these hormone pathways in the stress response.

### Osmotic stress- and salt-responsive regulatory genes in *Z. xanthoxylum* roots

**Fig. 3:**
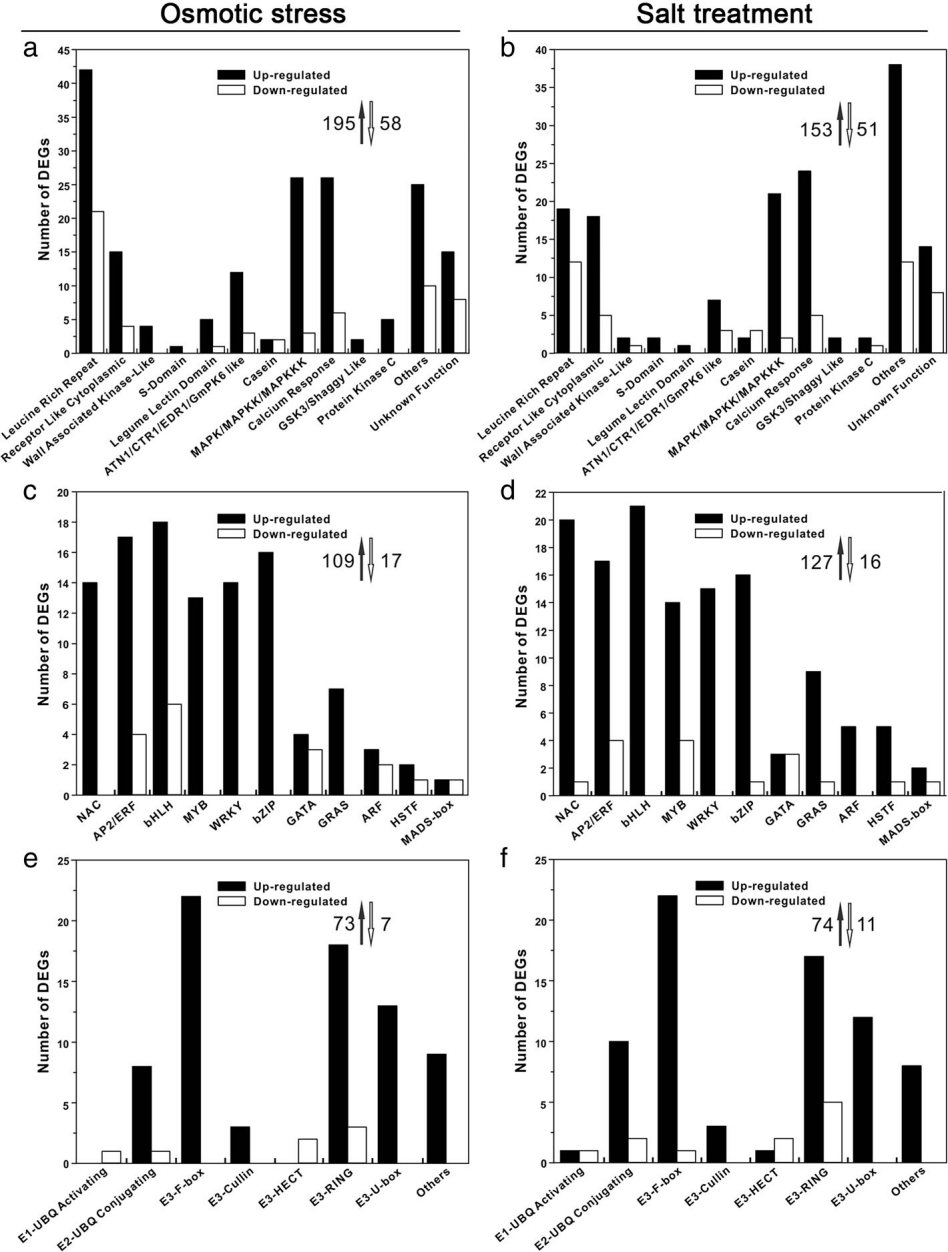
Distribution of osmotic stress- and salt-responsive DEGs encoding (**a, b**) protein kinases, (**c, d**) transcription factors, and (**e, f**) ubiquitin proteasome system-related enzymes in *Z. xanthoxylum* roots. Black bars, number of up-regulated genes. White bars, number of down-regulated genes. Black arrows, total number of up-regulated genes. White arrows, total number of down-regulated genes

Perception of stress signals leads to signal transduction and the activation of protective physiological and metabolic responses [12, 28]. To further analyze signal transduction genes in osmotic or salt-treated *Z. xanthoxylum* DEG libraries, we used Gene Ontology (GO) enrichment analysis. Over-represented categories were found to include protein kinases (253 and 204 kinase genes in osmotic-stressed and salt-treated DEGs libraries, respectively) (Fig. 3a and b), transcription factors (126 and 143 transcription factor genes in osmotic-stressed and salt-treated DEGs libraries, respectively) (Fig. 3c and d) and ubiquitin proteasome system (UPS) genes (80 and 85 UPS genes in osmotic-stressed and salt-treated DEGs libraries, respectively) (Fig. 3e and f).

#### Protein kinases

Protein kinases play key roles in the perception of stress-related signals. Major classes involved in signal perception are leucine-rich repeat receptor-like kinases (LRR-RLKs), cell-wall associated kinases, lectin-domain-containing receptor kinases, and receptor-like cytoplasmic kinases [29, 30]. A large number of these genes are rapidly induced in osmotic or salt stress treated Arabidopsis plants [30, 31]. Several have confirmed roles in drought and salt tolerance, including *FERRONIA* [32] and *RECEPTOR-LIKE PROTEIN KINASE 1* [33]. Numerous DEGs encoding RLKs are upregulated in our dataset (Fig. 3a and b and Additional file 3: Figure S2a). For example, the *Z. xanthoxylum FERRONIA*-like Unigene61098_All is up-regulated 4.6- and 7.5-fold during osmotic stress and salt treatment, respectively (Additional file 2: Table S3). Many RLK genes not previously linked to stress are also differentially upregulated in *Z. xanthoxylum* roots including LRR-RLK-like Unigene9800_All, Unigene176_All, and Unigene12756_All with highly significant blast hits to *Hevea brasiliensis BAK1* (*BRASSINOSTEROID INSENSITIVE 1-ASSOCIATED RECEPTOR KINASE 1*) [34], *Ricinus communis ERECTA* [35], and *Prunus avium BAM3* (*BARELY ANY MERISTEM 3*) [36], respectively (Additional file 2: Table S3). The Arabidopsis counterparts of these genes are well studied but not in the context of abiotic stress responses [34, 36–39]. Future work to evaluate the role of these genes in stress-related functions using Arabidopsis model plants is a valuable future direction.

Protein kinases are also important for signal transmission. Major classes include CDPKs (calcium-dependent protein kinases) and MAPKs (mitogen-activated protein kinases) [40]. In Arabidopsis, several CDPKs and MAPKs are involved in abiotic stress signaling pathways [41–43]. Arabidopsis plants overexpressing *CPK6* have enhanced tolerance to salt and drought stresses [42]. Similarly, transgenic barley (*Hordeum vulgare)* plants overexpressing *HvMPK1* showed greater tolerance to salt and other abiotic stresses [44]. Our DEG libraries include 31 and 28 predicted calcium response protein kinase genes, and 28 and 24 predicted MAPK genes in salt- and osmotic stress-treated *Z. xanthoxylum* roots, respectively (Fig. 3a and b, Additional file 3: Figure S2b and Additional file 2: Table S3). These data are consistent with known roles for Arabidopsis CDPKs and MAPKs in abiotic stress signaling.

#### Transcription factors

Transcription factors are important outputs of signaling pathways, directly responsible for the activation or repression of stress-responsive genes [1, 12, 45]. Major classes include NAC (NAM/ATAF/CUC), AP2/ERF (APETALA2 and ethylene-responsive element binding proteins), bHLH (basic helix-loop-helix), MYB (myeloblastosis), WRKY (WRKY-domain) and bZIP transcription factors whose members confer drought and salt stress tolerance in various plants [1]. For example, many rice WRKY genes exhibit obviously different functions under drought or salt stress [46] and soybean *WRKY54* over-expressed in transgenic Arabidopsis confers tolerance to drought and salt stress [47]. bZIP24 was identified by screening salt-inducible transcripts in Arabidopsis and a closely-related halophyte species with functional analysis showing that repression increased salt tolerance and the transcript abundance of numerous stress response genes [48]. Multiple bHLH transcription factors also have stress-related roles such as MYC2 (Myelocytomatosis protein 2) involved in jasmonic acid signaling and AIB (ABA-inducible bHLH-type transcription factor) whose overexpression enhances the drought tolerance of transgenic plants [49, 50]. Consistent with these studies, 126 and 144 transcription factors DEGs were identified in *Z. xanthoxylum* under osmotic stress and salt treatment, respectively. NAC, AP2/ERF, bHLH, MYB, WRKY, and bZIP families were the most abundant (Fig. 3c and d). Many of these DEGs matched to putative orthologs in other plants with a characterized role in stress biology. For example, CL8432.Contig3_All, CL6477.Contig2_All, Unigene2817_All, Unigene2222_All, Unigene2285_All were up-regulated in osmotic-stressed and salt-treated *Z. xanthoxylum* (Additional file 3: Figure S2c and Additional file 2: Table S4). These genes encode predicted orthologs of Arabidopsis ERF96 [51], bHLH106 [52], MYB3 [53], WRKY22 [54], and bZIP53 [55], which are previously identified as salt or drought responsive genes with important roles in abiotic stress signaling pathways.

Numerous other transcription factor genes with less-defined roles in abiotic stress were up- or down-regulated in osmotic-stressed and salt-treated *Z. xanthoxylum* roots (Fig. 3c and d and Additional file 2: Table S4). Examples include Unigene57160_All, Unigene16368_All, CL6534.Contig1_All and Unigene9789_All, which are orthologous to Arabidopsis MYB40, NAC083, NAC035, and WRKY69, respectively (Additional file 2: Table S4). Several transcription factor families not previously associated with abiotic stress mechanisms were also identified. Examples include GATA, GRAS (GAI/RGA/SCR) and ARF transcription factors. In rice, abiotic stress signaling by ABA and salt treatment causes the induction and differential splicing of 28 GATA transcription factor genes revealing tight regulation of transcript abundance and splice variants and possible diverse roles in abiotic stress signaling [56]. Within these families, the expression level of some genes changed significantly. For example, CL11183.Contig1_All predicted to encode a GRAS transcription factor and was up-regulated more than 10-fold in response to osmotic stress and salt treatment in *Z. xanthoxylum* (Additional file 2: Table S4). Some of these previously uncharacterized transcription factors might represent novel regulators of osmotic stress or salt tolerance.

#### UPS enzymes

Ubiquitination is an important mechanism for regulating protein turnover in response to stimuli and UPS enzymes play a central role [57, 58]. Accordingly, numerous *Z. xanthoxylum* DEGs induced under osmotic-stress and salt treatment encode UPS-related components such as ubiquitins, proteasome subunits, E1 ubiquitin-activating enzymes, E2 ubiquitin-conjugating enzymes, and E3 ubiquitin ligases (Fig. 3e and f and Additional file 3: Figure S2d). Among these, 22 F-box E3 genes were induced both in osmotic stress- and salt-treated *Z. xanthoxylum* (Fig. 3e and f). Four of these genes match to Arabidopsis orthologs with known roles in stress signaling including CL7586.Contig2_All (Additional file 2: Table S5). This F-box gene is a predicted ortholog of Arabidopsis *TUBBY LIKE PROTEIN3* important for plant responses to ABA, salt, and osmotic stress [59]. Several U-box, RING (really interesting new gene) and HECT (homology to E6-APC terminus) E3 ubiquitin ligase genes are also up- or down-regulated in osmotic stress- or salt-treated *Z. xanthoxylum* roots (Fig. 3e and f, Additional file 3: Figure S2d and Additional file 2: Table S5) including CL4067.Contig1_All. This gene is a predicted ortholog of Arabidopsis *PUB18* (Plant U-box 18) induced by ABA, drought and salt stress, which functions as a negative regulator of ABA-mediated drought stress responses [60]. These findings highlight potentially important roles for E3 ligases as both positive and negative stress response regulators in *Z. xanthoxylum* roots.

In summary, 459 and 432 protein kinase, transcription factor, and UPS genes were differentially expressed in osmotic-stressed and salt-treated *Z. xanthoxylum* DEG libraries, respectively. Hierarchical cluster analyses indicate that transcripts for most of these genes are increased at 6 h but not 24 h (Additional file 3: Figure S2). For example, 103 up-regulated and 41 down-regulated transcription factor genes were among the DEGs of *Z. xanthoxylum* roots treated for 6 h with salt (Additional file 3: Figure S2) but only 9 of these transcription factor genes were among DEGs at 24 h (Additional file 4: Figure S3). Thus, 6 h DEGs are more likely to be early response regulators in *Z. xanthoxylum* roots under osmotic stress and salt treatments. To test of the reproducibility of our RNA-sequencing data, we used qRT-PCR to independently monitor the transcript abundance of 20 randomly selected DEGs representing protein kinases, transcription factors, and UPS enzymes. These results were generally consistent with the RNA-sequencing data (Additional file 2: Table S6).

### Protein-protein interaction networks of Z. *xanthoxylum* in response to osmotic stress and salt treatments

**Fig. 4:**
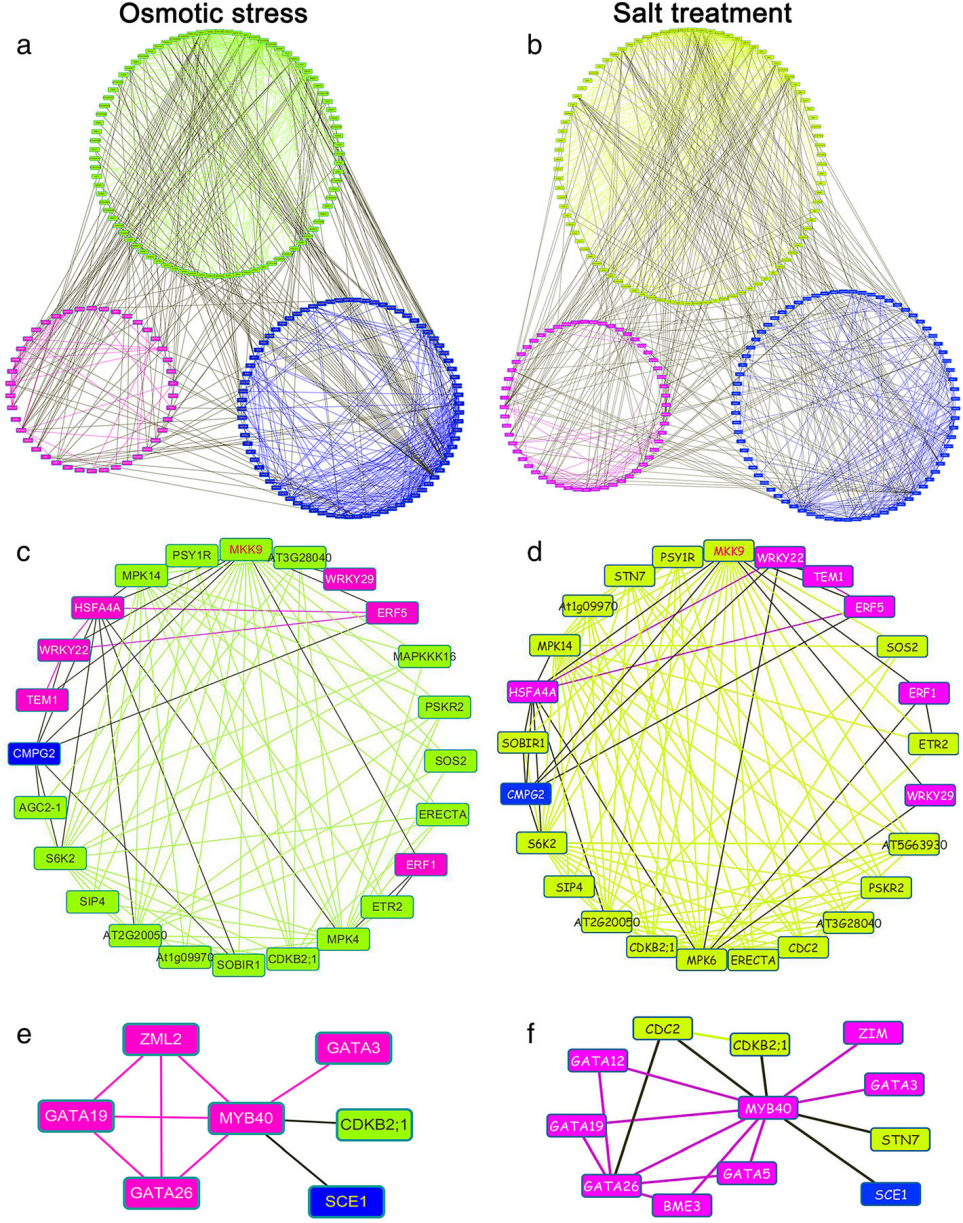
Protein-protein interaction analysis of Arabidopsis DEG orthologs. **a** osmotic stress-responsive DEG network and (**b**) salt-responsive DEG network. For high resolution images, see Additional file 5: Figure S4 and Additional file 6: Figure S5. **c,d** predicted MKK9 (Unigene22615_All) module showing regulatory proteins and relationships among them (nodes and edges) for *Z. xanthoxylum* roots in response to (**c**) osmotic stress and (**d**) salt, respectively. **e, f** predicted MYB40 (Unigene57160_All) module showing regulatory proteins and relationships among them (nodes and edges) for *Z. xanthoxylum* roots in response to (**e**) osmotic stress and (**f**) salt, respectively. Green nodes and edges (lines), kinases and interactions among them; magenta nodes and edges, transcription factors and interactions among them; blue nodes and edges, UPS enzymes and interactions among them; gray edges, interactions among kinases, transcription factors, and UPS enzymes except those among the same category of proteins

**Fig. 5:**
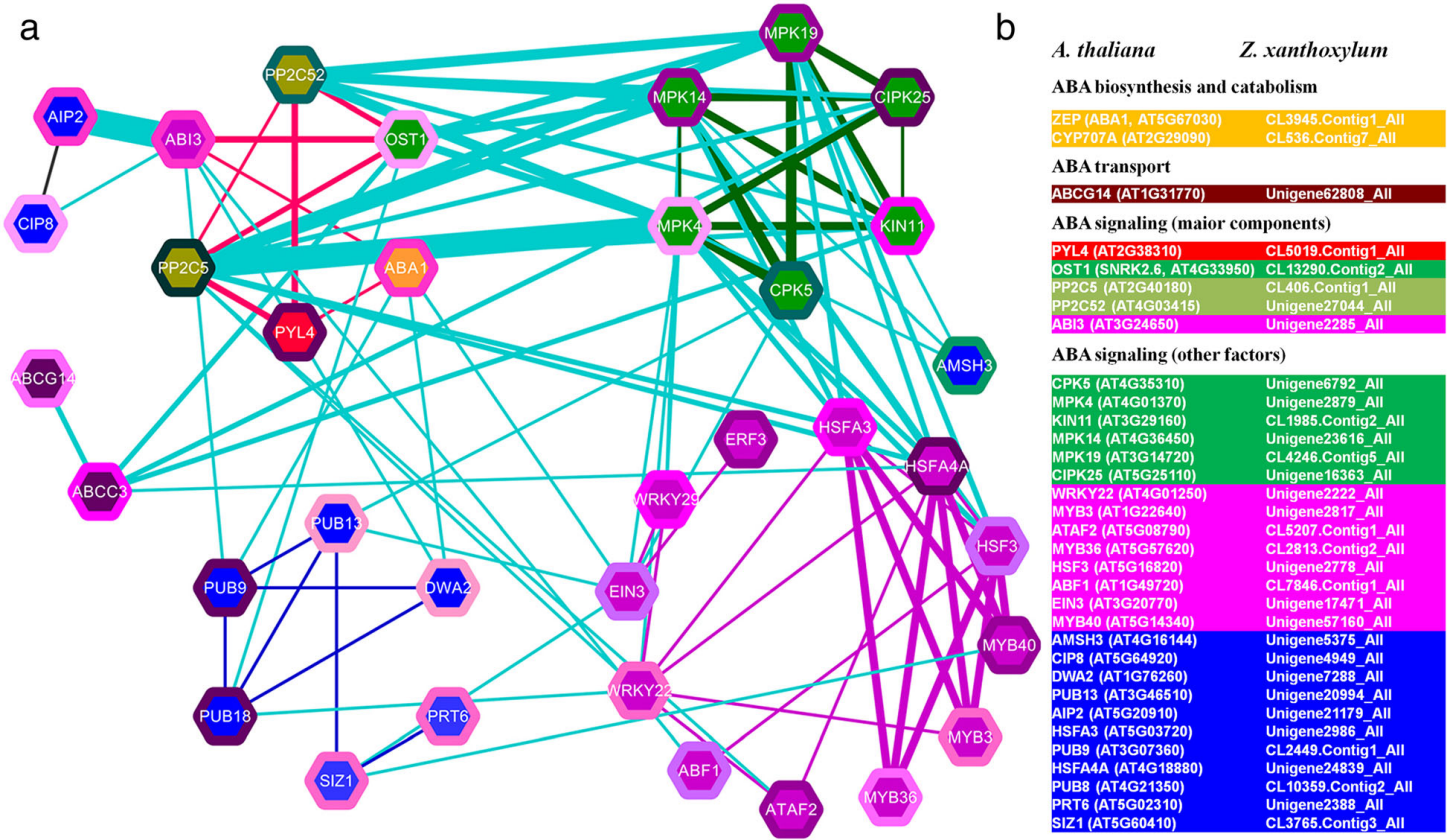
Module for ABA signaling pathway in osmotic stress responses of *Z. xanthoxylum* roots. **a** Functional network based on known Arabidopsis ABA-dependent signaling components, cross-referenced against the complete DEG functional network. Node colors represent different classes of signaling components: orange, ABA biosynthesis; red, receptors; yellow-green, PP2Cs, green, kinases; dark magenta, transporters; magenta, transcription factors; and blue, UPS enzymes. Border colors of nodes represent expression as log2 fold-change. Magenta, increased expression; the deeper the color, the higher the expression. Green, decreased expression; the deeper the color, the lower the expression. Edge (line) colors, interactions of proteins in the core ABA signaling pathway (red), kinases (green), UPS enzymes (blue), transcription factors (magenta), and proteins except in the same class (cyan). Thickness of edges, normalized link closeness; the thicker the edge, the closer the link. **b** ABA signaling and biosynthetic pathway components. Shading, colors represent different classes of signaling components: orange, ABA biosynthesis; red, receptors; yellow-green, PP2Cs, green, kinases; dark magenta, transporters; magenta, transcription factors; and blue, UPS enzymes

To further explore functional relationships among regulatory DEGs from *Z. xanthoxylum*, BLASTp homology searches against the Arabidopsis TAIR 10 genome release were used to identify putative orthologs. Among 459 osmotic stress-responsive regulatory DEGs in *Z. xanthoxylum*, 359 unique DEG orthologs were identified in Arabidopsis. Similarly, among the 432 salt-responsive regulatory DEGs in *Z. xanthoxylum*, 339 unique DEG orthologs were identified in Arabidopsis. STRING analysis was performed to present known gene co-expression, genetic interactions, protein interactions, and protein subcellular localization. Cytoscape was used to calculate and display the most over-represented functional categories, demonstrating a central gene cluster enriched in osmotic stress and salt responsive genes (Fig. 4a and b; Additional file 5: Figure S4 and Additional file 6: Figure S5). In the osmotic stress-responsive network, 220 DEG proteins, including 89 protein kinases, 55 transcription factors, and 76 UPS enzymes are represented (Fig. 4a, Additional file 5: Figure S4 and Additional file 2: Table S7). In the salt-responsive network, 204 DEG proteins, including 85 protein kinases, 42 transcription factors, and 77 UPS enzymes are represented (Fig. 4b, Additional file 6: Figure S5 and Additional file 2: Table S7). These highly integrated networks incorporated both conserved and novel gene products associated with exposure to osmotic stress and salt conditions in *Z. xanthoxylum*. For example, Unigene22615_All matches to Arabidopsis MKK9, a known MAPK determinant of salt tolerance [61]. When protein kinases, transcription factors, and UPS enzymes directly related to MKK9 are examined, these proteins together with relationships among them form a sub-network showing predicted regulatory events surrounding Unigene22615_All in response to osmotic-stress and salt treatment of *Z. xanthoxylum*, respectively (Fig. 4c and d). On the other hand, Unigene57160_All blast hits to functionally unresolved Arabidopsis transcription factor gene *MYB40*. Network analysis between MYB40 and protein kinase, transcription factors, and UPS enzymes reveals potentially novel regulatory events (Fig. 4e and f). For example, MYB40 may regulate plant response to osmotic and salt stress by interacting with GATA transcription factors, regulating the transcription level of GATA genes, or GATAs may function upstream of *MYB40* (Fig. 4e and f). These relationships provide a theoretical basis for investigating *MYB40* function in stress tolerance. Network analysis also reveals an extensive ABA signaling network in *Z. xanthoxylum* roots in response to osmotic-stress and salt treatments (Fig. 5 and Additional file 2: Table S1). Under osmotic stress, the majority of ABA pathway genes are up-regulated, including 2 ABA biosynthesis and catabolism genes, 1 transport gene, 1 receptor gene, 1 SnRK gene, 1 ABI transcription factor gene, and various other ABA responsive and UPS genes (Fig. 5b). In total, 30 interrelated proteins shed light on a complex ABA-dependent network (Fig. 5a and b). Analysis of conserved and novel gene products within these networks provides a foundation for future study (Figs. 4 and 5 and Additional file 2: Table S7).

### Validation of stress-related functions for novel *ZxNAC* regulatory genes

**Fig. 6:**
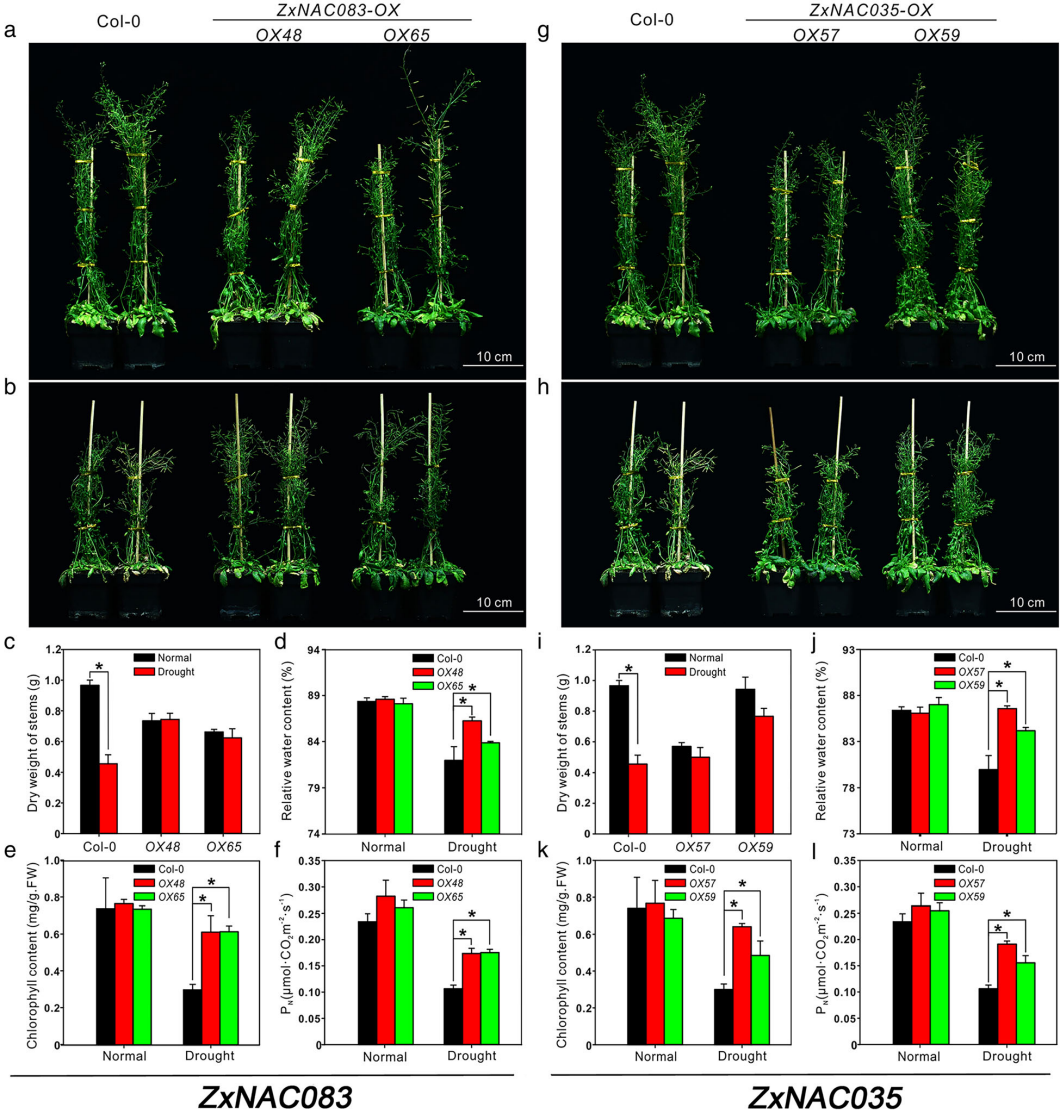
Phenotypes and physiological parameters of Col-0, *ZxNAC083* and *ZxNAC035* overexpression transgenic plants under normal conditions and period drought stress. Two independent lines were analyzed for each transgenic. Representative images are shown. **a, g** Col-0, *ZxNAC083* and *ZxNAC035* overexpression transgenic plants under normal conditions. **b, h** Col-0, *ZxNAC083* and *ZxNAC035* overexpression transgenic plants under period drought stress (2-weeks water withheld, 1-week recovery). **c, d, e, f** Physiological parameters of Col-0 and *ZxNAC083* overexpression transgenic lines under normal conditions and period drought stress. **c** Dry weight of stems, (**d**) relative water content, (**e**) chlorophyll content, and (**f**) net photosynthetic rate. **i, j, k, l** Physiological parameters of Col-0 and *ZxNAC035* overexpression transgenic plants under normal conditions and period drought stress. **i** Dry weight of stems, (**j**) relative water content, (**k**) chlorophyll content, and (**l**) net photosynthetic rate. Data are mean ± SD of three replicates. **c,i** Asterisks, significant difference. *p* < 0.05 (t-test). (**d, e, f, j, k, l**) Asterisks, significant difference. *p* < 0.05 (Duncan’s test)

To further analyze the biological relevance of our dataset, novel *Z. xanothxylum* regulatory DEGs from our study were tested as potential osmotic and salt stress determinants in Arabidopsis. Eight candidate genes were selected for functional characterization including two protein kinases genes [Unigene9800_All (Zx*SERK1*) and CL11556.Contig3_All (*ZxMRH1*)] and six transcription factor genes [(Unigene16368_All (*ZxNAC083*), CL6534.Contig1_All (*ZxNAC035*), Unigene57160_All (*ZxMYB40*), CL9880.Contig2_All (*ZxWRKY29*), Unigene9789_All (*ZxWRKY69*) and Unigene2222_All (*ZxWRKY22*)]. Results are presented for *ZxNAC083* and *ZxNAC03*5, named after their putative orthologs in Arabidopsis. Independent qRT-PCR analysis confirmed that both genes are highly induced in osmotic-stressed and salt-treated *Z. xanthoxylum* roots (Additional file 7: Figure S6a, b and Additional file 2: Table S6). Transgenic Arabidopsis plants expressing these genes under the control of a strong constitutive Cauliflower Mosaic Virus 35S promoter were next generated. Two independent transgenic lines per construct were selected for phenotypic analyses under normal, drought, and salt-stress conditions (Additional file 7: Figure S6c, d). The drought tolerance of wild type Col-0 and transgenic lines was first compared by withholding water for 7 days [62]. Under this treatment, wild-type plants began to wilt but the *ZxNAC083* and *ZxNAC035* transgenic plants grew well (Additional file 8: Figure S7). To further analyze growth and physiological parameters, period drought stress was applied by withholding water for 2 weeks followed by normal watering for 7 days [63]. The wild-type treated Arabidopsis plants had etiolated and wilted leaves, whereas the transgenic lines grew well (Fig. 6a, b, g, and h). To quantify effects on growth, the dry weight of stems was measured. Under normal watering conditions, transgenic plants had slightly shorter stems with a lower dry weight compared to wild-type plants (Fig. 6a, c, g, and i). However, wild-type plant growth was more inhibited by drought compared to transgenic plants (Fig. 6b, c, h, and i). The dry weight of stems from transgenic plants under control and drought stress conditions was similar (Fig. 6c and i). Physiological parameters including relative water content, chlorophyll content, and net photosynthetic rate were also measured. Under well-watered conditions, wild-type and transgenic plants exhibited no significant differences (Fig. 6d and j). After period drought treatment, the relative water content of transgenic plants was higher than wild-type Arabidopsis, suggesting that *ZxNAC083* and *ZxNAC035* can regulate osmotic homeostasis in plants under drought conditions (Fig. 6d and j). Water deficit can lead to a decrease in chlorophyll content thus lowering net photosynthetic rate [63, 64]. The chlorophyll content of transgenic plants was maintained at more normal levels compared to Col-0 plants (Fig. 6e and k). Transgenic plants also showed a higher net photosynthetic rate compared to Col-0 under drought treatment (Fig. 6f and l).

**Fig. 7:**
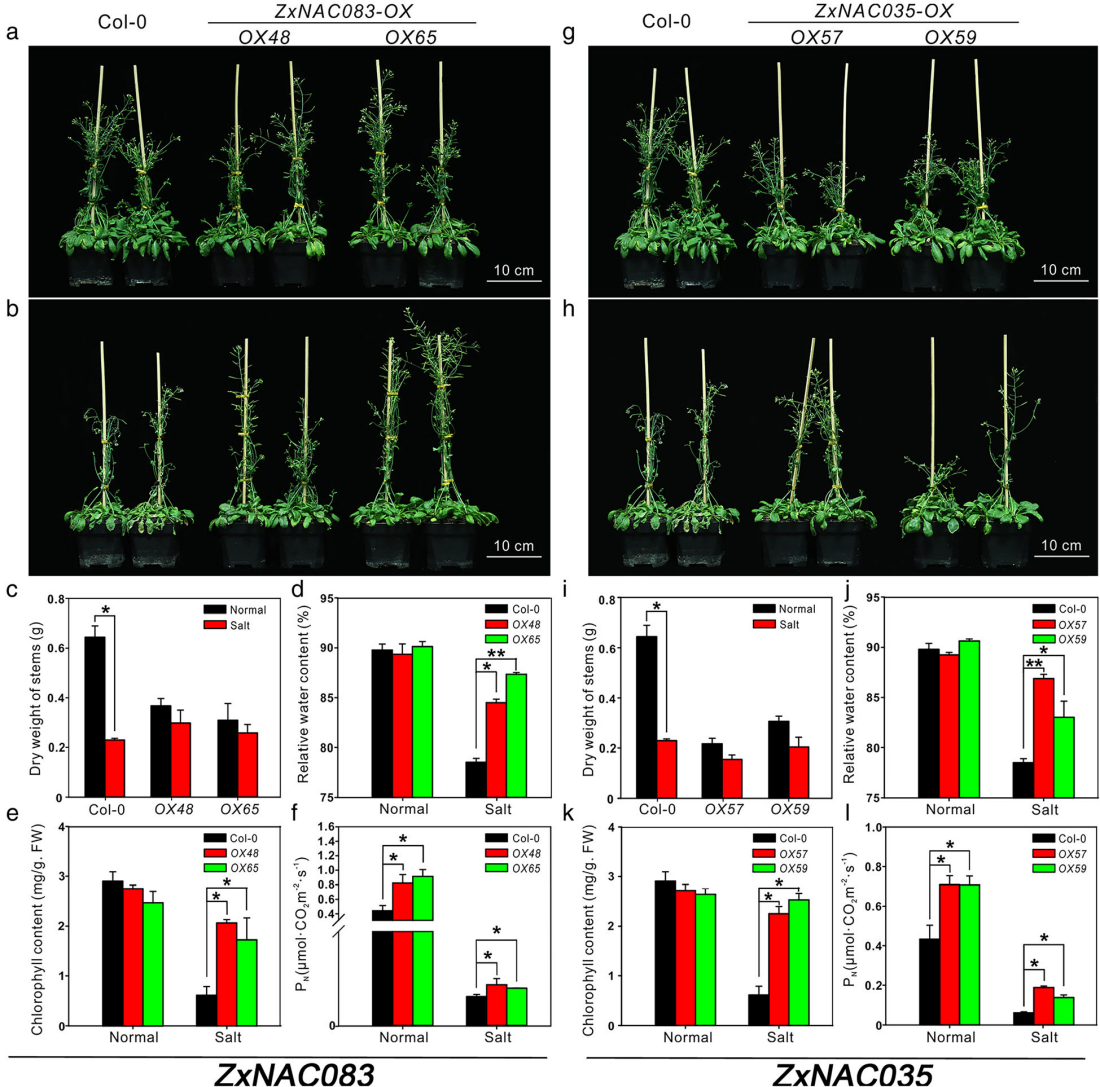
Phenotypes and physiological parameters of Col-0, *ZxNAC083* and *ZxNAC035* overexpression transgenic plants under normal condition and salt stress. Two independent lines were analyzed for each transgenic. Representative images are shown. **a, g** Col-0, *ZxNAC083* and *ZxNAC035* overexpression transgenic plants under normal condition. **b, h** Col-0, *ZxNAC083* and *ZxNAC035* overexpression transgenic plants under salt stress (2-weeks, 100 mM NaCl). **c, d, e, f** Physiological parameters of Col-0, *ZxNAC083* overexpression transgenic plants under normal conditions and salt stress. **c** Dry weight of stems, **d** relative water content, **e** chlorophyll content, and (**f**) net photosynthetic rate. **i, j, k, l** Physiological parameters of Col-0, *ZxNAC035* overexpression transgenic plants under normal conditions and salt stress. **i** Dry weight of stems, (**j**) relative water content, (**k**) chlorophyll content, and (**l**) net photosynthetic rate. Data are mean ± SD of three replicates. **c, i** Asterisks, significant difference. *p* < 0.05 (t-test). (**d, e, f, j, k, l**) Asterisks, significant difference. *p* < 0.05 (Duncan’s test)

Finally, we compared the salinity tolerance of wild type and transgenic lines. Plants were irrigated with 100 mM NaCl solution for 2 weeks. After treatment, the rosette leaves of Col-0 were faded and withered whereas transgenic plants grew well (Fig. 7b and h). Col-0 plants had longer stems with a greater dry weight compared to transgenic plants under normal conditions (Fig. 7a and g). However, wild-type plant growth was more severely inhibited by salt stress compared to transgenic plants (Fig. 7c and i). Physiological parameters including relative water content, chlorophyll content and net photosynthetic rate were also measured. Salinity can decrease soil water potential leading to physiological drought. While the relative water content of wild-type and transgenic plants was similar under normal conditions, *ZxNAC083* and *ZxNAC035* overexpression lines showed significantly higher relative water content compared to wild-type plants after salt treatment (Fig. 7d and j). High relative water content improves chlorophyll stability and photosynthetic system integrity under physiological drought. Under salt stress, both chlorophyll content and net photsynthetic rate of *ZxNAC83* and *ZxNAC035* transgenic lines were significantly higher than Col-0 wild-type showing a protective effect (Fig. 7e, f, k, and l).

These combined data demonstrate that overexpression of *ZxNAC083* and *ZxNAC035* enhances Arabidopsis drought and salt tolerance. These data provide powerful evidence that regulatory genes identified in this study include authentic osmotic and salt stress determinants in *Z. xanthoxylum*. Many of these genes have unresolved functions in stress tolerance.

## Conclusions

This study provides a first comprehensive analysis of regulatory gene transcripts in *Z. xanthoxylum* under osmotic stress and salt treatments. These data reveal a complex network of conserved and unique regulatory genes induced in *Z. xanthoxylum* roots with potential roles in abiotic stress tolerance. Functional analysis of these genes is a rich resource for crop improvement.

## Methods

### Data acquisition and differential expression analysis

The transcriptomic data and digital gene expression libraries (C6, C24, D6, D24, S6, and S24) analyzed in this study were generated as previously described [20]. Seeds of *Z. xanthoxylum* used in this work were collected from wild plants in Alxa League of Inner-Mongolia Autonomous Region, China, and were stored in the Key Laboratory of Grassland Livestock Industry Innovation, Ministry of Agriculture and Rural Affairs, Lanzhou, China. The raw data are submitted to the NCBI Sequence Read Archive (PRJNA512400). Each transcriptomic library was constructed from seedling roots, using 5–6 seedlings per sample. Libraries C6 and C24 correspond to treatment with a mock solution (1/2 strength Hoagland nutrient solution) for 6 h and 24 h, respectively; D6 and D24 correspond to treatment with an osmotic stress solution (1/2 strength Hoagland nutrient solution with added sorbitol, equivalent to − 0.5 MPa osmotic potential, sorbitol concentration ≈ 165 mM) for 6 h and 24 h, respectively; and S6 and S4 correspond to treatment with a salt solution (1/2 strength Hoagland nutrient solution with 50 mM NaCl) for 6 h and 24 h, respectively. Libraries were constructed using a tag-based digital gene expression protocol [20]. The resulting libraries were sequenced in parallel using an Illumina HiSeq™ 2000 sequencing platform (BGI Shenzhen). Low quality tags from each library were removed (e.g. tags with unknown nucleotide “N”, “empty tags” with no tag sequence between the adaptors, and tags with only one copy number). The resulting “clean” tags were mapped to our transcriptome reference database [20]. To establish differential expression, the number of clean tags for each gene was calculated and normalized to the number of transcripts per million clean tags [65]. To compare differences in gene expression, the tag frequency in each library was statistically analyzed according to Audic and Claverie [66]. The false discovery rate (FDR) was used to determine the threshold *p-*value in multiple tests [65, 67]. Genes that displayed FDR<0.001 and absolute value of the log2 ratio>1 were selected as significantly different.

### DEGs regulatory pathways analysis, hierarchical clustering, and validation by qRT-PCR

The Blast2GO program (https://www.blast2go.com/) was employed to obtain Gene Ontology (GO) annotations for Unigenes. Regulatory pathways were investigated by matching *Z. xanthophyllum* genes to putative orthologs in the Kyoto Encyclopedia of Genes and Genomes (KEGG) protein database (www.genome.jp/dbget/). Hierchical cluster was performed using Heml software (http://hemi.biocuckoo.org/down.php). Default options were used and Pearson correlations were performed on gene expression data.

RNA sequencing data were verified by measuring the transcript abundance of randomly-selected predicted regulatory genes using total RNA extracted from treated roots treated as described above [20]. First-strand cDNA was synthesized from 2 μg of DNase-treated RNA according to the manufacturer’s instructions (TaKaRa Biotechnology, China). qRT-PCR was performed in triplicate on three bioreplicates using *Power* SYBR™ Green Master Mix (TaKaRa Biotechnology, China) on a StepOne Real-Time PCR Thermocycler (Applied Biosystems). To normalize sample variance, *ZxACTIN* (GenBank accession no. EU019550) was used as the internal control gene. The relative expression level of each gene were determined using the 2^-ΔΔCt^ method [65]. Primer-BLAST (https://www.ncbi.nlm.nih.gov/tools/primer-blast) was used for primer design. A standard curve and efficiency test were performed for each set of primers (Additional file 2: Table S8).

### Functional data mining in Arabidopsis and network analysis

*Z. xanthoxylum* DEGs were matched to putative orthologs in Arabidopsis by BLASTp analysis (*E*-value of 1e^− 6^) against the reference Arabidopsis TAIR10 genome annotation (http://www.arabidopsis.org). Protein-protein interaction analysis of protein function, protein subcellular localization, and gene co-expression of putative Arabidopsis orthologs was carried out in STRING [68] (http://www.string-db.org/). STRING protein-protein interaction images were re-modulated in Cytoscape [69] (https://cytoscape.org/).

### Generating overexpression lines

The coding regions of Unigene16368_All (*ZxNAC083*) and CL6534.Contig1_All (*ZxNAC035*) were amplified from seedling total RNA using a SMART RACE cDNA Amplification Kit (TaKaRa Biotechnology, China). The resulting cDNA products were cloned into pDONR™/Zeo using a Gateway® BP reaction (ThermoFisher Scientific, China) then inserted into binary vector pBIB-BASTA-35S-GWR-GFP using a Gateway® LR reaction (ThermoFisher Scientific, China). The resulting constructs were introduced into *Agrobacterium tumefaciens* strain GV3101 and used to transform wild-type Arabidopsis Col-0 plants by floral dipping (seeds of wild-type Arabidopsis Col-0 were obtained from Ministry of Education Key Laboratory of Cell Activities and Stress Adaptation, Lanzhou, China) [70]. Transgenic overexpression lines were validated using semi-quantitative RT-PCR to measure *ZxNAC083* and *ZxNAC035* transcript levels. *AtACTIN2* was used as the internal control gene. Primer sequences are provided in Additional file 2: Table S8.

### Phenotypic and physiological assessments of transgenic plants

Phenotypic assays were performed on Arabidopsis wild-type Col-0 and T3 homozygous transgenic overexpression lines using two independent lines per construct (*ZxNAC083-OX48*, *OX65* and *ZxNAC035-OX57*, *OX59*). 4-week-old plants grown on soil in pots (8 × 8 × 8 cm^3^) were subjected to drought or salt stress treatments as described with minor changes [71]. For period drought stress treatments, water was withheld for 2 weeks followed by normal watering for 7 days to permit recovery [71]. Under normal conditions, each pot was given 150 mL of water every 2 days. For salt stress treatments, each pot was given 150 mL of 100 mM NaCl solution every 2 days for 2 weeks. The dry weight of stems (shoots excluding rosette leaves), relative water content, chlorophyll content, and net photosynthetic rate were determined as previously described [19]. Seedlings were grown in a greenhouse at 20–22 °C with relative humidity 65–75% under long days (16 h light/8 h dark, photon flux density 100–120 μmol·m^− 2^·s^− 1^).^.^

## Additional files

Additional file 1: **Figure S1.** Venn diagrams showing DEGs in *Z. xanthoxylum* roots under osmotic stress and salt treatments. Yellow and green colors, up-regulated and down-regulated transcripts under osmotic stress for (a) 6 h and (b) 24 h, respectively. Red and blue colors, up-regulated and down-regulated transcripts under salt treatment for (a) 6 h and (b) 24 h, respectively. (JPG 257 kb)

Additional file 2: **Table S1.** ABA signaling pathway DEGs in *Z. xanthoxylum* roots under osmotic stress and salt treatment. **Table S2.** Auxin signaling pathway DEGs in *Z. xanthoxylum* roots under osmotic stress and salt treatments. **Table S3.** Selection and categorization of significant *Z. xanthoxylum* kinase DEGs in response to osmotic stress and salt treatments. **Table S4.** Selection and categorization of significant *Z. xanthoxylum* transcription factor DEGs in response to osmotic stress and salt treatments. **Table S5.** Selection and categorization of significant *Z. xanthoxylum* UPS enzyme DEGs in response to osmotic stress and salt treatments. **Table S6.** RNA seq data verification by qRT-PCR measurement of randomly selected *Z. xanthoxylum* osmotic stress and salt responsive protein kinase, transcription factor, and UPS enzyme genes. **Table S7.** DEGs matched to predicted Arabidopsis orthologs in the complete gene networks in *Z. xanthoxylum* roots under osmotic stress and salt treatment. Yes (Y) or No (N) indicate gene representation in the corresponding DEG libraries. **Table S8.** Primers used in current study . (DOCX 91 kb)

Additional file 3: **Figure S2.** Hierarchical cluster analysis of differentially expressed genes encoding (a) receptor like kinases, (b) Ca^2+^ related kinases, (c) E3 ubiquitin ligases, and (d) transcription factors in *Z. xanthoxylum* roots under osmotic stress and salt treatments. Unigenes are matched to Arabidopsis orthologs where possible. (JPG 12062 kb)

Additional file 4: **Figure S3.** Hierarchical cluster analysis of transcription factor genes that are differentially expressed in *Z. xanthoxylum* roots at both 6 h and 24 h under salt treatment. Unigenes are matched to Arabidopsis orthologs where possible. (JPG 963 kb)

Additional file 5: **Figure S4.** High resolution images of Fig. 4a. (PDF 28 kb)

Additional file 6: **Figure S5.** High resolution images of Fig. 4b. (PDF 33 kb)

Additional file 7: **Figure S6.** Expression analysis of *ZxNAC083* and *ZxNAC035*. (a-b) qRT-PCR validation of RNA sequencing data in *Z. xanthoxylum* roots under osmotic stress or salt treatment for 6 h. (c) Semi-quantitative RT-PCR assay showing overexpression of *ZxNAC083* in transgenic Arabidopsis plants compared to Col-0 wild-type. *ZxNAC083* (20 cycles) and *AtActin2* (19 cycles). (d) Semi-quantiative RT-PCR experiment assay showing overexpression of *ZxNAC035* in transgenic Arabidopsis plants compared to Col-0 wildtype. *ZxNAC035* (25 cycles) and *AtActin2* (19 cycles). (JPG 429 kb)

Additional file 8: **Figure S7.** Phenotypes of Col-0, *ZxNAC083* and *ZxNAC035* overexpression transgenic plants under normal conditions and 7-day drought stress at vegetative phases. Two independent lines were analyzed for each transgenic. Representative images are shown. (a,c) Col-0, *ZxNAC083* and *ZxNAC035* overexpression transgenic plants under normal conditions. (b,d) Col-0, *ZxNAC083* and *ZxNAC035* overexpression transgenic plants under 7-day drought stress. (JPG 1069 kb)

## Abbreviations

ABA: Abscisic acid
AIB: ABA-inducible bHLH-type transcription factor
AP2/ERF: APETALA2 and ethylene-responsive element binding proteins
ARF: Auxin Response Factor
AUX/IAA: Auxin/Indole-3-Acetic Acid
*BAK1*: *BRASSINOSTEROID INSENSITIVE 1-ASSOCIATED RECEPTOR KINASE 1*
*BAM3*: *BARELY ANY MERISTEM 3*
bHLH: Basic helix-loop-helix
bZIP: Basic leucine zipper
CDPKs: Calcium-dependent protein kinases
DEGs: Differentially expressed genes
FDR: False discovery rate
GH3: Gretchen Hagen 3
GO: Gene Ontology
GRAS: GAI/RGA/SCR
HECT: Homology to E6-APC terminus
KEGG: Kyoto Encyclopedia of Genes and Genomes
LRR-RLKs: Leucine-rich repeat receptor-like kinases
MAPKs: Mitogen-activated protein kinases
MYB: Myeloblastosis
MYC2: Myelocytomatosis protein 2
NAC: NAM/ATAF/CUC
PP2C: Protein phosphatase 2C
PUB18: Plant U-box 18
PYL/PYR/RCAR: PYRABACTIN RESISTANCE1/PYR1-LIKE/REGULATORY COMPONENTS OF ABA RECEPTORS
RING: Really interesting new gene
ROS: Reactive oxygen species
SAUR: Small Auxin-Up RNA
SnRKs: Sucrose non-fermenting like kinases
TIR1/AFB: Transport Inhibitor Response1/Auxin Signaling F-box
UPS: Ubiquitin proteasome system
WRKY: WRKY-domain
*Z. xanthoxylum*: *Zygophyllum xanthoxylum*
