# Supplementary figures and images for: Transcriptome analysis reveals regulatory framework for salt and osmotic tolerance in a succulent xerophyte
Source: BMC Plant Biol. 2019 Feb 28;19:88. doi: 10.1186/s12870-019-1686-1 (PMC6394007; doi:10.1186/s12870-019-1686-1)

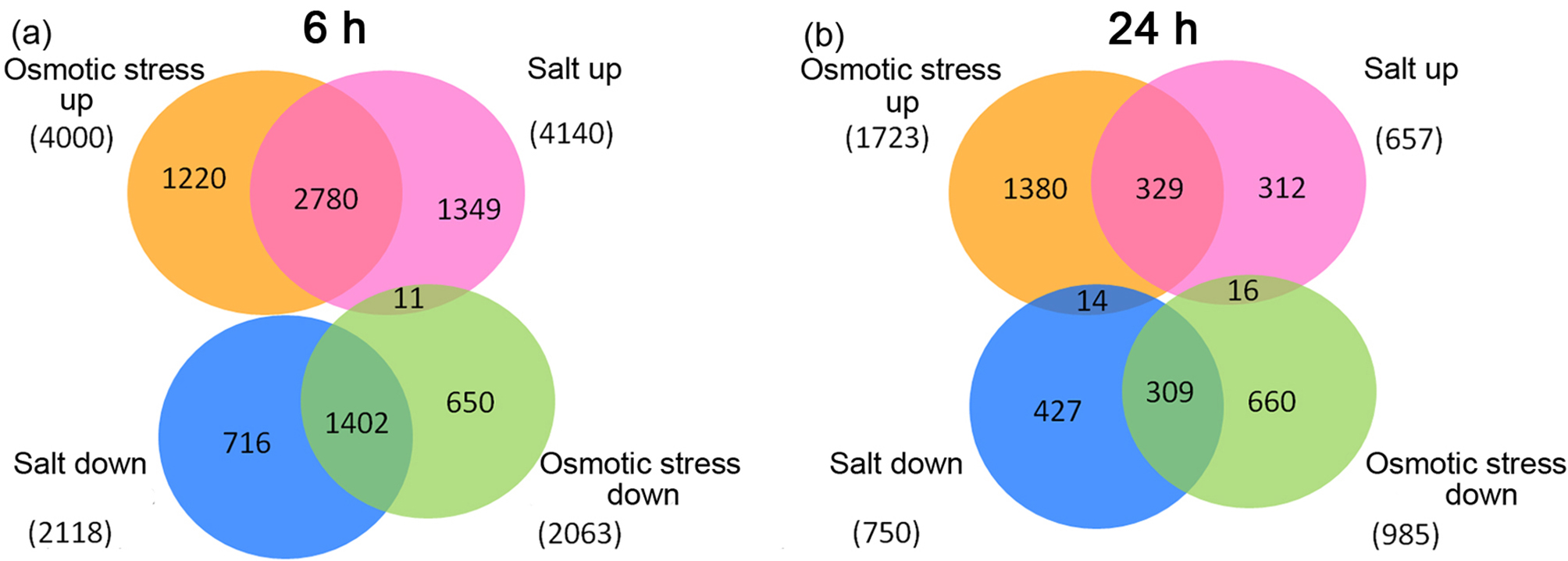

Supplement: Supplementary file 1 — Figure S1. Venn diagrams showing DEGs in Z. xanthoxylum roots under osmotic stress and salt treatments. Yellow and green colors, up-regulated and down-regulated transcripts under osmotic stress for (a) 6 h and (b) 24 h, respectively. Red and blue colors, up-regulated and down-regulated transcripts under salt treatment for (a) 6 h and (b) 24 h, respectively. (JPG 257 kb) [file 12870_2019_1686_MOESM1_ESM.jpg]

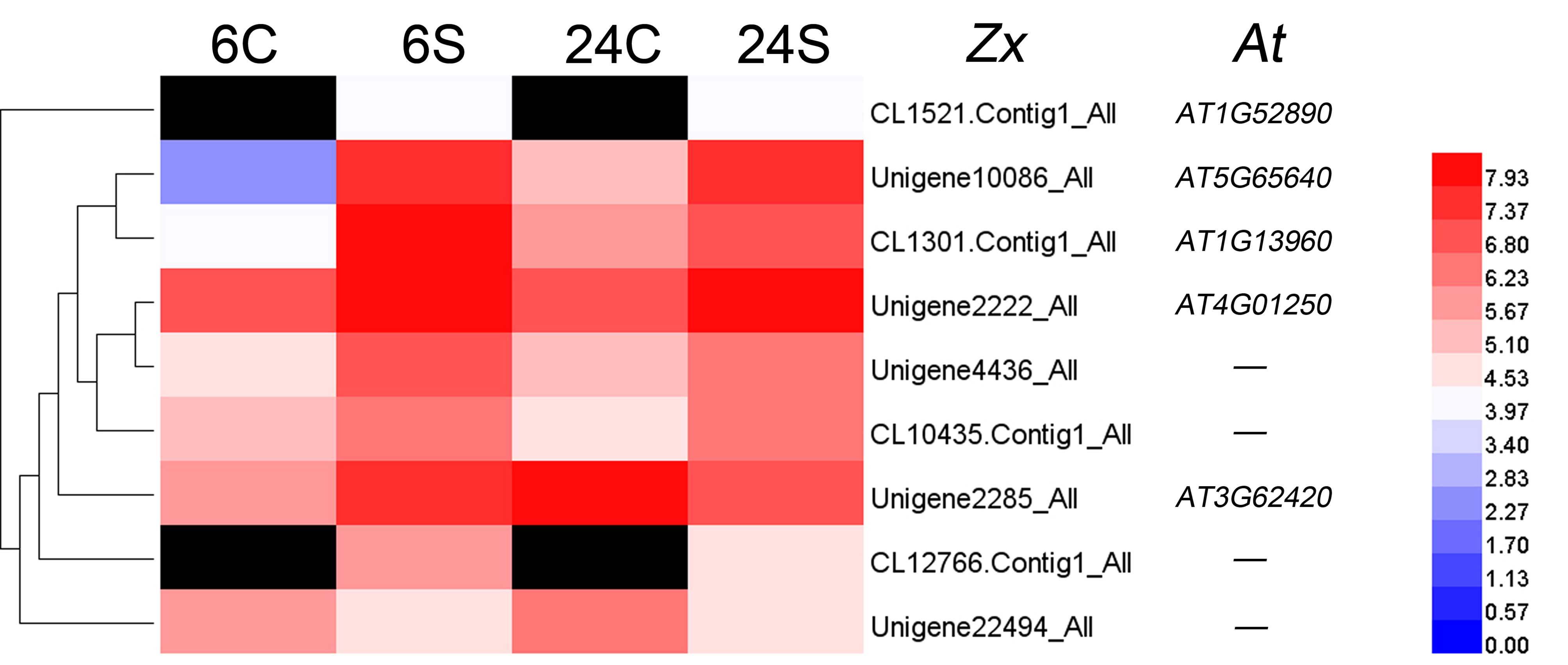

Supplement: Supplementary file 4 — Figure S3. Hierarchical cluster analysis of transcription factor genes that are differentially expressed in Z. xanthoxylum roots at both 6 h and 24 h under salt treatment. Unigenes are matched to Arabidopsis orthologs where possible. (JPG 963 kb) [file 12870_2019_1686_MOESM4_ESM.jpg]

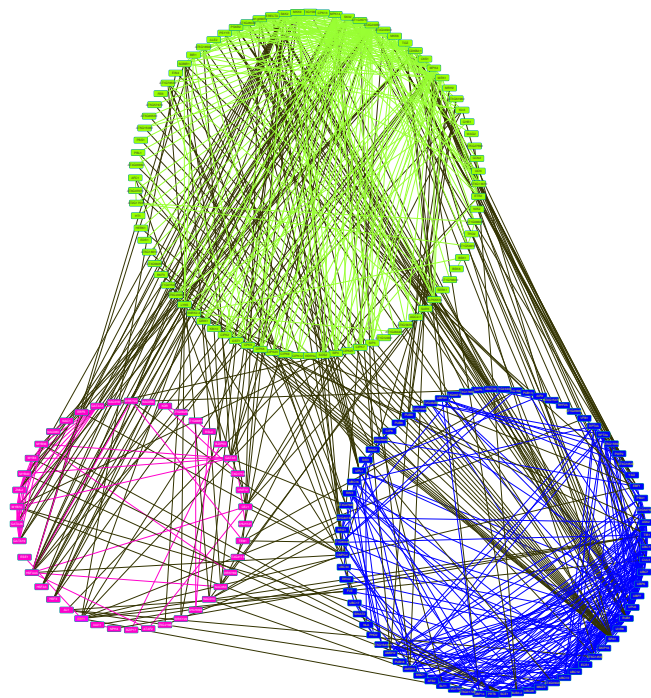

Supplement: Supplementary file 5 — Figure S4. High resolution images of Fig. 4a. (PDF 28 kb) [file 12870_2019_1686_MOESM5_ESM.pdf]

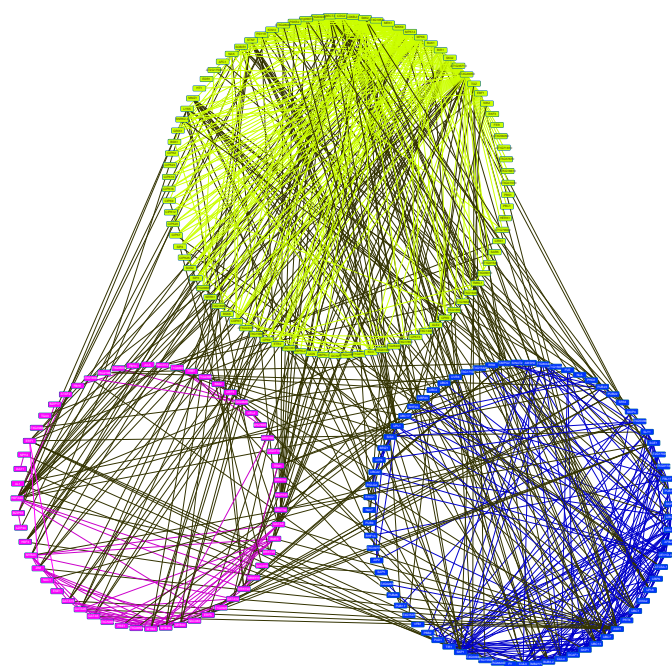

Supplement: Supplementary file 6 — Figure S5. High resolution images of Fig. 4b. (PDF 33 kb) [file 12870_2019_1686_MOESM6_ESM.pdf]

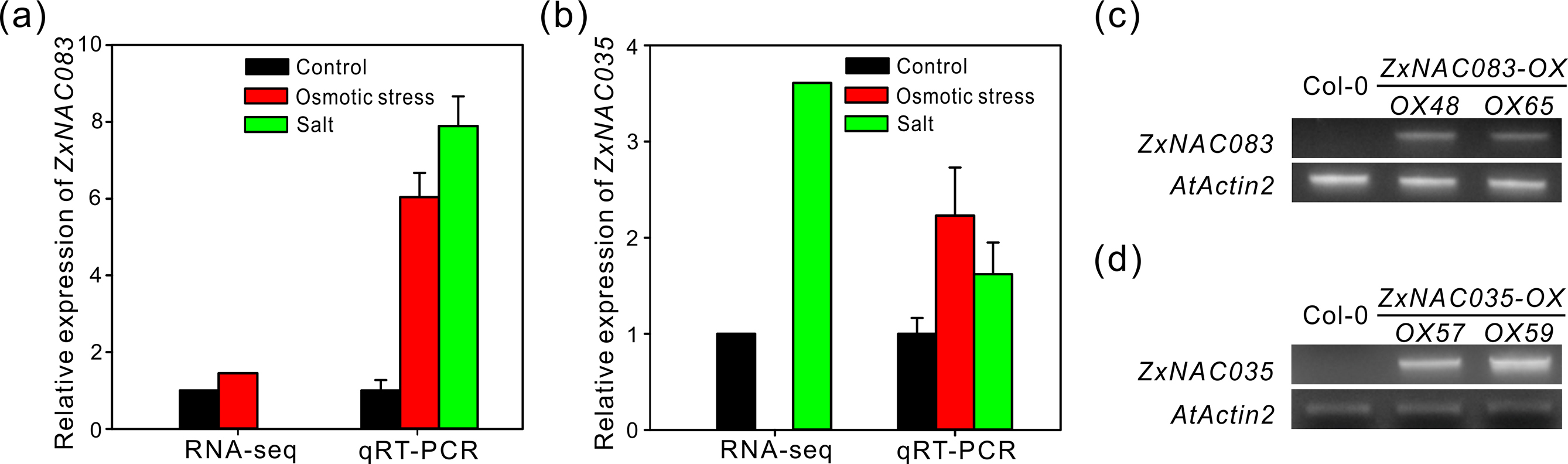

Supplement: Supplementary file 7 — Figure S6. Expression analysis of ZxNAC083 and ZxNAC035. (a-b) qRT-PCR validation of RNA sequencing data in Z. xanthoxylum roots under osmotic stress or salt treatment for 6 h. (c) Semi-quantitative RT-PCR assay showing overexpression of ZxNAC083 in transgenic Arabidopsis plants compared to Col-0 wild-type. ZxNAC083 (20 cycles) and AtActin2 (19 cycles). (d) Semi-quantiative RT-PCR experiment assay showing overexpression of ZxNAC035 in transgenic Arabidopsis plants compared to Col-0 wildtype. ZxNAC035 (25 cycles) and AtActin2 (19 cycles). (JPG 429 kb) [file 12870_2019_1686_MOESM7_ESM.jpg]

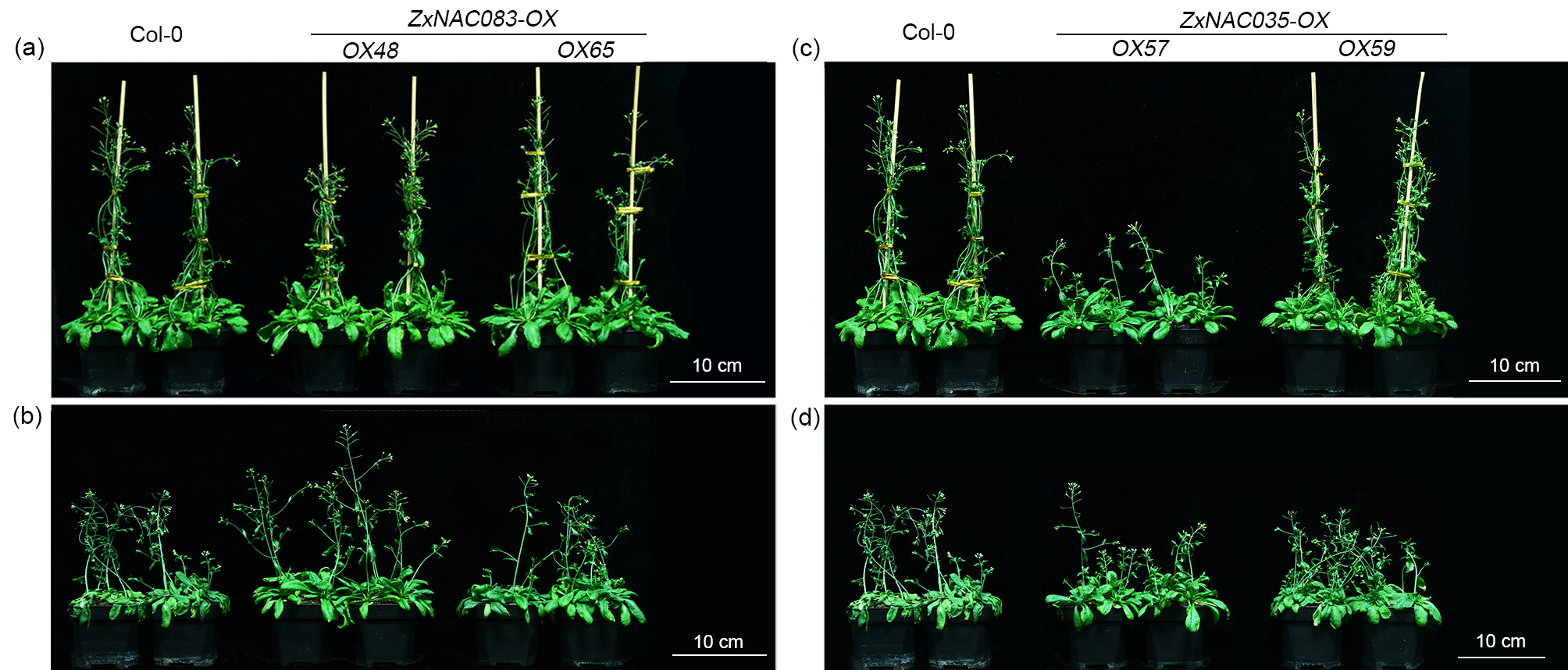

Supplement: Supplementary file 8 — Figure S7. Phenotypes of Col-0, ZxNAC083 and ZxNAC035 overexpression transgenic plants under normal conditions and 7-day drought stress at vegetative phases. Two independent lines were analyzed for each transgenic. Representative images are shown. (a,c) Col-0, ZxNAC083 and ZxNAC035 overexpression transgenic plants under normal conditions. (b,d) Col-0, ZxNAC083 and ZxNAC035 overexpression transgenic plants under 7-day drought stress. (JPG 1069 kb) [file 12870_2019_1686_MOESM8_ESM.jpg]
