# Supplementary material for: Transcriptome analysis reveals regulatory framework for salt and osmotic tolerance in a succulent xerophyte
Source: BMC Plant Biol. 2019 Feb 28;19:88. doi: 10.1186/s12870-019-1686-1 (PMC6394007; doi:10.1186/s12870-019-1686-1)
Supplement: Supplementary file 2 — Table S1. ABA signaling pathway DEGs in Z. xanthoxylum roots under osmotic stress and salt treatment. Table S2. Auxin signaling pathway DEGs in Z. xanthoxylum roots under osmotic stress and salt treatments. Table S3. Selection and categorization of significant Z. xanthoxylum kinase DEGs in response to osmotic stress and salt treatments. Table S4. Selection and categorization of significant Z. xanthoxylum transcription factor DEGs in response to osmotic stress and salt treatments. Table S5. Selection and categorization of significant Z. xanthoxylum UPS enzyme DEGs in response to osmotic stress and salt treatments. Table S6. RNA seq data verification by qRT-PCR measurement of randomly selected Z. xanthoxylum osmotic stress and salt responsive protein kinase, transcription factor, and UPS enzyme genes. Table S7. DEGs matched to predicted Arabidopsis orthologs in the complete gene networks in Z. xanthoxylum roots under osmotic stress and salt treatment. Yes (Y) or No (N) indicate gene representation in the corresponding DEG libraries. Table S8. Primers used in current study . (DOCX 91 kb) [file 12870_2019_1686_MOESM2_ESM.docx]

**Additional file 2**

**Table S1.** ABA signaling pathway DEGs in *Z. xanthoxylum* roots under osmotic stress and salt treatment.

| **Contig ID** | **RNA-Seq**  **Log2 Ratio (DR6/CR6)** | **RNA-Seq**  **log2 Ratio (SR6/CR6)** |
| --- | --- | --- |
| **PYR/PYL family** | | |
| Unigene40727_All | 4.2 | 3.0 |
| CL5019.Contig1_All | 2.8 | 2.6 |
| Unigene23274_All | 2.5 | 2.3 |
| Unigene13774_All | 1.5 | 1.6 |
| CL10799.Contig1_All | － | 1.4 |
| **PP2C** |  |  |
| Unigene19369_All | 4.3 | － |
| Unigene7395_All | 3.0 | 2.2 |
| Unigene8130_All | 2.8 | － |
| CL472.Contig1_All | 2.4 | 3.0 |
| CL350.Contig6_All | － | 3.1 |
| CL2300.Contig1_All | 1.9 | 1.9 |
| CL46.Contig2_All | 1.8 | 1.9 |
| CL13301.Contig1_All | － | 1.3 |
| Unigene12346_All | 1.4 | 1.1 |
| Unigene797_All | 1.2 | 1.0 |
| CL2558.Contig1_All | -3.1 | － |
| Unigene27044_All | -2.3 | -2.9 |
| CL406.Contig1_All | -1.5 | － |
| **SnRK2** | | |
| CL13290.Contig2_All | 2.9 | 2.6 |
| Unigene9954_All | 1.4 | 1.2 |
| Unigene5144_All | 1.2 | － |
| Unigene60647_All | -1.2 | -1.9 |
| **ABF** |  |  |
| CL4322.Contig2_All | 3.4 | 3.0 |
| CL4322.Contig1_All | 2.9 | 2.1 |
| Unigene2285_All | 2.6 | 2.6 |
| CL7846.Contig1_All | 1.8 | － |
| CL9888.Contig2_All | 1.4 | 1.5 |
| CL1422.Contig1_All | 1.1 | － |

**Table S2.** Auxin signaling pathway DEGs in *Z. xanthoxylum* roots under osmotic stress and salt treatments.

| **Contig ID** | **RNA-Seq**  **Log2 Ratio (DR6/CR6)** | **RNA-Seq**  **log2 Ratio (SR6/CR6)** |
| --- | --- | --- |
| **TIR1/ABF receptor family** | | |
| Unigene5528_All | 2.4 | 2.7 |
| Unigene2651_All | 2.1 | 2.1 |
| **AUX/IAA** | | |
| CL3960.Contig2_All | 8.2 | ­－ |
| CL7248.Contig2_All | 7.6 | － |
| Unigene6598_All | － | 7.8 |
| Unigene4769_All | 2.8 | 3.2 |
| CL1569.Contig2_All | 2.6 | － |
| CL3690.Contig1_All | 2.4 | 1.4 |
| CL10102.Contig1_All | 2.2 | 1.1 |
| CL1569.Contig1_All | 2.1 | 1.6 |
| CL11533.Contig1_All | 1.9 | 1.3 |
| Unigene7218_All | 1.8 | 1.2 |
| CL3690.Contig2_All | 1.6 | － |
| Unigene15695_All | 1.5 | 1.2 |
| CL4279.Contig2_All | 1.3 | － |
| Unigene47230_All | -8.2 | － |
| CL4279.Contig4_All | -1.2 | -2.7 |
| **ARF transcription factor** | | |
| CL9800.Contig1_All | 8.3 | 7.6 |
| CL5492.Contig2_All | 2.1 | 1.6 |
| CL3453.Contig1_All | 1.8 | 1.3 |
| Unigene7765_All | － | 1.1 |
| Unigene46888_All | -2.9 | － |
| Unigene5051_All | -1.1 | － |
| **GH3 family** | | |
| CL10990.Contig2_All | － | 8.1 |
| Unigene2674_All | 2.7 | 1.7 |
| CL8542.Contig2_All | 2.7 | 2.7 |
| CL3620.Contig3_All | 1.6 | 1.4 |
| Unigene29333_All | -7.6 | -7.6 |
| **SAUR family** | | |
| Unigene45869_All | － | 8.5 |
| Unigene7192_All | 7.8 | － |
| Unigene40989_All | 5.3 | 6.0 |
| Unigene16411_All | 3.0 | 3.5 |
| Unigene19731_All | 2.7 | 2.5 |
| Unigene12630_All | 2.1 | 2.5 |
| Unigene59994_All | 2.0 | － |
| Unigene5167_All | 1.7 | － |
| Unigene5191_All | 1.1 | 1.0 |
| Unigene29963_All | -1.4 | -3.9 |

**Table S3.** Selection and categorization of significant *Z. xanthoxylum* protein kinase DEGs in response to osmotic stress and salt treatments. The reference information comes from TAIR (<https://www.arabidopsis.org/>).

| **Contig ID** | **Control** | **Drought**  **6 h** | **salt**  **6 h** | **Putative**  **Arabidopsis ortholog** | **Abiotic stress relative function** | **Reference** |
| --- | --- | --- | --- | --- | --- | --- |
| **Receptor like kinase (RLK)** | | | | | | |
| Unigene61098_All | 15 | 85 | 127 | *FER* | Positively regulate abiotic stress responses | [32] |
| Unigene18100_All | 49 | 191 | 131 | *RKF3* | Unknown |  |
| Unigene5204_All | 30 | 284 | 38 | [*HT1*](http://www.arabidopsis.org/servlets/TairObject?id=29149&type=locus) | Essential for stomatal movement | [72] |
| CL5798.Contig4_All | 10 | 38 | 119 | *[EDR1](http://www.arabidopsis.org/servlets/TairObject?id=29149&type=locus)* | Unknown | [37,38] |
| Unigene9800_All | 61 | 135 | 179 | *SERK1* | Unknown |  |
| Unigene176_All | 38 | 7 | 12 | *ERECTA* | Unknown | [39] |
| CL3059.Contig2_All | 15 | 172 | 266 | *HSL1* | Unknown | [73] |
| Unigene12756_All | 44 | 116 | 124 | *BAM3* | Unknown | [36] |
| Unigene4893_All | 2 | 38 | 54 | *AT3G47570* | Unknown |  |
| Unigene6580_All | 35 | 122 | 93 | *AT1G01540* | Unknown |  |
| CL1102.Contig3_All | 53 | 121 | 180 | *AT5G15080* | Unknown |  |
| CL11556.Contig3_All | 0 | 66 | 65 | *MRH1* | Unknown |  |
| Unigene21737_All | 43 | 204 | 223 | *LECRK-S.5* | Unknown |  |
| Unigene8229_All | 9 |  | 65 | *LECRK-S.4* | Unknown |  |
| Unigene13249_All | 79 |  | 199 | *LECRK-S.1* | Unknown |  |
| **Mitogen-activated protein kinase (MAPK)** | | | | | | |
| CL7742.Contig2_All | 170 | 609 | 476 | *MEK1* | Positive regulator of plant resistance to drought and salt stress | [41] |
| CL3131.Contig1_All | 4 | 30 | 30 | *MKK2* | Regulates MPK6 and MPK4 in response to cold and salt stresses | [74] |
| Unigene22615_All | 17 | 232 | 247 | *MKK9* | Negativey regulates plant resistance to salt stress | [61] |
| Unigene6580_All | 35 | 122 | 93 | *AT1G01540* | Unknown |  |
| CL1102.Contig3_All | 53 | 121 | 180 | *AT5G15080* | Unknown |  |
| **Calcium dependent protein kinase (CDPK) and CBL-interacting protein kinase (CIPK)** | | | | | | |
| CL5.Contig3_All | 87 | 299 | 404 | *CIPK23* | Controls ion homeostasis and abiotic stress | [75] |
| Unigene16363_All | 4 | 65 | 34 | *CIPK25* | Drought and heat stress responsive | [76] |
| CL12723.Contig1_All | 18 |  | 70 | *CPK6* | Positively regulates plant resistance to drought and salt stress | [42] |
| Unigene12466_All | 148 | 417 | 354 | *CPK4* | Positive regulator of ABA signaling | [77] |
| CL7369.Contig3_All | 6 | 47 | 44 | *CPK13* | Essential for stomatal movement | [78] |
| CL10984.Contig1_All | 4 | 79 | 77 | *CIPK5* | Positively regulates plant resistance to salt stress | [76] |
| CL7369.Contig2_All | 378 | 146 | 87 | *CPK2* | Unknown |  |
| CL3288.Contig2_All | 91 | 245 | 460 | *CIPK10* | Unknown |  |
| CL311.Contig2_All | 25 | 202 | 203 | *CIPK26* | Unknown |  |

**Table S4.** Selection and categorization of significant *Zygophyllum xanthoxylum* transcription factor DEGs in response to osmotic stress and salt treatments. The reference information comes from TAIR (<https://www.arabidopsis.org/>).

| **Contig ID** | **Control** | **Drought**  **6 h** | **salt**  **6 h** | **Putative**  **Arabidopsis ortholog** | **Abiotic stress relative function** | **Reference** |
| --- | --- | --- | --- | --- | --- | --- |
| **NAC** | | | | | |  |
| CL1521.Contig1_All | 0 | 27 | 16 | *ANAC019* | Positively regulates resistance to heat stress | [79] |
| Unigene34418_All | 54 | 717 | 281 | *ANAC100* | Unknown |  |
| CL5207.Contig1_All | 12 | 74 | 45 | *ANAC081* | Expression is induced by ABA | [80] |
| Unigene8038_All | 155 | 456 | 537 | *ANAC083* | Unknown |  |
| CL6534.Contig1_All | 2 | 2 | 26 | *ANAC035* | Unknown |  |
| CL169.Contig1_All | 0 | 34 | 18 | *NAC014* | Unknown |  |
| **AP2/ERF** | | | | | |  |
| CL7054.Contig2_All | 0 | 43 | 49 | *EDF1* | Unknown |  |
| CL8432.Contig3_All | 14 | 215 | 190 | *ERF95* | Unknown |  |
| CL7054.Contig1_All | 200 | 939 | 606 | *AT1G64380* | dehydration stress memory genes in Arabidopsis | [81] |
| **MYB** | | | | | |  |
| Unigene2817_All | 81 | 250 | 224 | *MYB3* | Unknown |  |
| Unigene57160_All | 6 | 38 | 51 | *MYB40* | Unknown |  |
| Unigene13937_All | 314 | 1374 | 777 | *AT1G19000* | Unknown |  |
| CL2813.Contig2_All | 74 | 244 | 231 | *MYB36* | Unknown |  |
| Unigene18742_All | 18 | 2 | 2 | *TRB1* | Unknown |  |
| **WRKY** | | | | | |  |
| Unigene2222_All | 120 | 366 | 314 | *WRKY22* | Unknown |  |
| CL9880.Contig2_All | 0 | 21 | 32 | *WRKY29* | Unknown |  |
| CL11642.Contig2_All | 2 | 33 | 26 | *WRKY31* | Unknown |  |
| Unigene9789_All | 36 | 519 | 189 | *WRKY69* | Unknown |  |
| Unigene9786_All | 9 | 126 | 77 | *WRKY35* | Unknown |  |
| Unigene752_All | 0 | 23 | 25 | *WRKY11* | Unknown | [82] |
| **Others** | | | | | |  |
| CL6477.Contig2_All | 40 | 144 | 115 | *bHLH106* | Negatively regulate plant resistance to ABA and abiotic stress | [52] |
| Unigene2285_All | 69 | 277 | 213 | *bZIP53* | Negatively regulate plant resistance to ABA and salt stress | [55] |
| Unigene1938_All | 115 | 473 | 314 | *AIF3* | Unknown |  |
| CL11183.Contig1_All | 39 | 1016 | 447 | *AT5G48150* | Unknown |  |
| Unigene205_All | 48 | 123 | 113 | *AT4G39790* | Unknown |  |

**Table S5.** Selection and categorization of significant *Zygophyllum xanthoxylum* UPS enzyme DEGs in response to osmotic stress and salt treatments. The reference information comes from TAIR (<https://www.arabidopsis.org/>).

| **Contig ID** | **Control** | | **Drought**  **6 h** | | **salt**  **6 h** | | **Putative**  **Arabidopsis ortholog** | | **Abiotic stress relative function** | **Reference** | |  |
| --- | --- | --- | --- | --- | --- | --- | --- | --- | --- | --- | --- | --- |
| **F-box protein** | | | | | | | | | | |  | |
| CL7586.Contig2_All | | 0 | | 17 | | 30 | | *TLP3* | Response to ABA and osmotic stress | | [59] | |
| Unigene5528_All | | 23 | | 131 | | 158 | | *TIR1* | Negatively regulates oxidative and salt stress tolerance | | [27] | |
| Unigene17402_All | | 29 | | 437 | | 185 | | *KMD1* | Unknown | |  | |
| CL10877.Contig3_All | | 30 | | 153 | | 132 | | *AT1G55270* | Unknown | |  | |
| Unigene9538_All | | 85 | | 335 | | 323 | | *KMD2* | Unknown | |  | |
| Unigene24229_All | | 21 | | 3 | | 3 | | *AT5G01720* | Unknown | |  | |
| Unigene14425_All | | 10 | | 56 | | 58 | | *VFB1* | Unknown | |  | |
| CL3743.Contig2_All | | 42 | | 306 | | 223 | | *AT2G17020* | Unknown | |  | |
| **RING finger E3 ubiquitin ligase** | | | | | | | | | | |  | |
| Unigene21179_All | | 77 | | 335 | | 259 | | *AIP2* | Negatively regulates ABA, salt and drought stress signaling | | [83] | |
| Unigene782_All | | 69 | | 417 | | 267 | | *AT5G42200* | Unknown | |  | |
| Unigene17354_All | | 45 | | 7 | | 10 | | *AT4G14220* | Unknown | |  | |
| Unigene2448_All | | 87 | | 1346 | | 885 | | *RMA3* | Unknown | |  | |
| CL1594.Contig1_All | | 72 | | 162 | | 161 | | *RIN3* | Unknown | |  | |
| Unigene10187_All | | 32 | | 123 | | 94 | | *BAH1* | Unknown | |  | |
| CL11499.Contig3_All | | 47 | | 5 | | 3 | | *AT2G42160* | Unknown | |  | |
| Unigene21619_All | | 9 | | 74 | | 40 | | *SINAT2* | Unknown | |  | |
| **U-box protein** | | | | | | | | | | |  | |
| CL2449.Contig1_All | | 4 | | 30 | | 25 | | *PUB9* | Negative regulation of ABA signaling | | [84] | |
| Unigene19482_All | | 5 | | 78 | | 65 | | *PUB33* | Unknown | |  | |
| CL5577.Contig2_All | | 33 | | 220 | | 219 | | *PUB26* | Unknown | |  | |
| CL4067.Contig1_All | | 64 | | 256 | | 212 | | *PUB18* | Unknown | | [60] | |
| CL9071.Contig2_All | | 53 | | 191 | | 220 | | *PUB8* | Unknown | |  | |
| **E2** | | | | | | | | | | |  | |
| Unigene6798_All | | 335 | | 147 | | 99 | | *UBC7* |  | |  | |
| Unigene445_All | | 0 | | 18 | | 15 | | *UBC13* |  | |  | |
| **SUMO ligase** | | | | | | | | | | |  | |
| Unigene18868_All | | 107 | | 404 | | 307 | | *AHUS5* |  | |  | |

**Table S6.** RNA-seq data verification by qRT-PCR measurement of randomly selected *Z. Xanthoxylum* osmotic stress and salt responsive protein kinase, transcription factor, and UPS enzyme genes. “__” means no corresponding ortholog in Arabidopsis. Data represent mean ± SD were calculated from three replicates. Three independent biological replicates were performed and consistent results were obtained. Only one representative set of data is shown.

| ***Z. xanthoxylum*** | **Log2 Ratio (DR6/CR6)**  **RNA-seq qRT-PCR** | | **log2 Ratio (SR6/CR6)**  **RNA-seq qRT-PCR** | | ***A. thaliana*** |
| --- | --- | --- | --- | --- | --- |
| **Protein kinase** | | | | | |
| CL4246.Contig5_All | 2.66 | 1.70±0.10 | 2.90 | 2.50±0.18 | *MPK13* |
| Unigene22003_All | 2.50 | 1.51±0.09 | 2.06 | 2.51±0.42 | *BIR1* |
| Unigene22615_All | 3.65 | 1.86±0.08 | 3.75 | 2.10±0.15 | *MKK9* |
| Unigene23616_All | 2.45 | 1.70±0.11 | 2.03 | 2.23±0.10 | *MPK1* |
| Unigene61098_All | 2.38 | 1.58±0.80 | 2.97 | 23.32±2.09 | *FER* |
| CL8091.Contig1_All | 4.74 | 4.10±0.36 | 3.60 | 7.33±0.25 | *­­__* |
| **Transcription factor** | | | | | |
| Unigene16368_All | 1.45 | 6.03±0.63 | － | 7.88±0.77 | *NAC083* |
| CL2554.Contig3_All | 3.54 | 6.12±0.79 | 4.11 | 8.64±0.13 | *NAC100* |
| CL6534.Contig1_All |  | 2.23±0.50 | 3.61 | 1.62±0.33 | *NAC035* |
| Unigene20426_All | 3.59 | 4.58±0.72 | 2.28 | 7.20±0.44 | *ERF5* |
| Unigene57160_All | 2.54 | 10.38±0.05 | 2.98 | 4.28±0.00 | *MYB43* |
| Unigene14772_All | 3.72 | 4.91±0.51 | 4.51 | 7.63±0.53 | *WRKY65* |
| Unigene9789_All | 3.73 | 2.13±0.47 | 2.28 | 2.76±0.39 | *WRKY7* |
| CL9880.Contig2_All | 8.04 | 5.01±1.07 | 8.67 | 6.00±1.36 | *WRKY29* |
| **UPS enzyme** | | | | | |
| Unigene17402_All | 3.79 | 5.05±0.58 | 2.56 | 8.03±0.69 | *KMD1* |
| Unigene5528_All | 2.38 | 1.26±0.19 | 2.67 | 1.94±0.21 | *TIR1* |
| CL5577.Contig2_All | 2.62 | 1.42±0.63 | 2.62 | 2.09±0.16 | *PUB26* |
| Unigene5077_All | 2.21 | 2.12±0.19 | 2.15 | 2.11±0.14 | *UBC10* |
| Unigene19871_All | 2.32 | 2.01±0.33 | 2.13 | 3.48±0.26 | *E2 17* |
| Unigene10187_All | 4.38 | 1.0±0.88 | 1.44 | 6.18±0.28 | *NLA* |

**Table S7.** DEGs matched to predicted Arabidopsis orthologs in the complete gene networks in *Z. xanthoxylum* roots under osmotic stress and salt treatment. Yes (Y) or No (N) indicate gene representation in the corresponding DEG libraries.

| ***Z.xanthoxylum***  **Contig ID** | **Putative**  **Arabidopsis orthologs** | **Osmotic stress** | **Salt** |
| --- | --- | --- | --- |
| **Protein Kinase** | | | |
| Unigene16363_All | AT5G25110 (CIPK25) | Y | Y |
| Unigene17634_All | AT2G30360 (SIP4) | Y | Y |
| CL311.Contig2_All | AT5G35410(SOS2) | Y | Y |
| Unigene19845_All | AT3G49660 (WDR5a) | Y | N |
| CL5673.Contig1_All | AT4G18700 (CIPK12) | Y | N |
| Unigene61644_All | AT4G18700 (CIPK12) | Y | Y |
| CL4951.Contig1_All | AT1G01140 (CIPK9) | Y | Y |
| CL5.Contig3_All | AT1G30270 (CIPK23) | Y | Y |
| Unigene6792_All | AT4G35310 (CPK5) | Y | Y |
| Unigene5144_All | AT4G33950 (OST1) | Y | N |
| CL3288.Contig4_All | AT5G07070 (CIPK2) | Y | Y |
| Unigene4368_All | AT3G01090 (KIN10) | Y | Y |
| CL6872.Contig4_All | AT3G45240 (GRIK1) | Y | Y |
| Unigene9954_All | AT1G10940 (SNRK2.4 ) | Y | Y |
| Unigene6865_All | AT2G33580 (LYK5) | Y | Y |
| Unigene5265_All | AT5G20050 | Y | N |
| Unigene6580_All | AT1G01540 | Y | Y |
| CL3498.Contig2_All | AT2G48010 (RKF3) | Y | Y |
| CL11556.Contig3_All | AT3G56050 | Y | Y |
| CL4709.Contig2_All | AT5G18610 | Y | Y |
| CL5798.Contig4_All | AT1G08720 (EDR1) | Y | Y |
| CL11024.Contig2_All | AT3G46290 (HERK1) | Y | Y |
| Unigene5204_All | AT1G62400 (HT1) | Y | Y |
| Unigene14586_All | AT2G17530 | Y | Y |
| Unigene10000_All | AT3G07070 | Y | Y |
| Unigene60167_All | AT3G53570 (AFC1) | Y | Y |
| CL5729.Contig5_All | AT4G00960 | Y | Y |
| Unigene15142_All | AT3G55450 (PBL1) | Y | Y |
| Unigene11511_All | AT5G13160 (PBS1) | Y | Y |
| CL1102.Contig3_All | AT5G15080 | Y | Y |
| Unigene60047_All | AT5G65530 | Y | N |
| Unigene21842_All | AT5G57670 | Y | Y |
| Unigene61098_All | AT3G51550 (FER) | Y | Y |
| CL6222.Contig3_All | AT1G78530 | Y | Y |
| CL940.Contig1_All | AT5G47230 | Y | Y |
| Unigene22003_All | AT2G31880 (SOBIR1) | Y | Y |
| CL6393.Contig1_All | AT5G48380 (BIR1) | Y | Y |
| Unigene62796_All | AT5G18500 | Y | Y |
| CL11451.Contig1_All | AT2G20300 (ALE2) | Y | Y |
| CL6001.Contig1_All | AT1G72300 (PSY1R) | Y | Y |
| Unigene2540_All | AT5G53890 (PSKR2) | Y | Y |
| CL995.Contig2_All | AT3G28040 | Y | Y |
| CL3059.Contig2_All | AT1G09970 | Y | Y |
| Unigene176_All | AT2G26330 (ERECTA) | Y | Y |
| Unigene18881_All | AT3G08720 (S6K2) | Y | Y |
| Unigene22615_All | AT1G73500(MKK9) | Y | Y |
| CL3540.Contig1_All | AT3G15890 | Y | Y |
| CL7369.Contig1_All | AT3G51850 (CPK13) | Y | Y |
| Unigene23616_All | AT4G36450 (MPK14) | Y | Y |
| CL3589.Contig1_All | AT4G00720 (SK32) | Y | Y |
| CL6969.Contig3_All | AT1G35710 | Y | Y |
| Unigene32_All | AT2G20050 | Y | Y |
| CL9874.Contig2_All | AT4G04940 | Y | Y |
| Unigene35564_All | AT5G56580 (MKK6) | Y | N |
| Unigene5014_All | AT5G16750 (TOZ) | Y | Y |
| CL9409.Contig3_All | AT1G76540 (CDKB2;1 ) | Y | Y |
| Unigene16191_All | AT2G27960 (CKS1) | Y | N |
| Unigene2879_All | AT4G01370 (MPK4) | Y | N |
| Unigene17377_All | AT4G26070 (MEK1 ) | Y | Y |
| CL9527.Contig2_All | AT3G61960 | Y | Y |
| CL12797.Contig1_All | AT2G17220 (Kin3) | Y | Y |
| Unigene12228_All | AT4G20940 (GHR1) | Y | Y |
| Unigene22479_All | AT3G14370 (WAG2) | Y | N |
| CL2926.Contig2_All | AT5G27550 | Y | Y |
| Unigene16959_All | AT3G59410 (GCN2) | Y | Y |
| Unigene419_All | AT1G51170 (UCN) | Y | Y |
| CL6250.Contig1_All | AT3G13670 | Y | Y |
| Unigene12086_All | AT3G23150 (ETR2) | Y | Y |
| Unigene7420_All | AT3G25560 (NIK2) | Y | Y |
| CL1915.Contig1_All | AT1G09600 | Y | Y |
| CL6596.Contig2_All | AT1G24706 (THO2) | Y | N |
| CL12559.Contig2_All | AT1G54610 | Y | N |
| Unigene16222_All | AT4G35230 (BSK1) | Y | Y |
| CL9457.Contig2_All | AT5G41260 (BSK8) | Y | Y |
| CL2382.Contig1_All | AT1G79600 | Y | Y |
| Unigene9800_All | AT1G71830 (SERK1) | Y | Y |
| CL2585.Contig1_All | AT4G26890 (MAPKKK16) | Y | N |
| Unigene4237_All | AT4G30520 (SARK) | Y | Y |
| CL11195.Contig1_All | AT3G25250 (AGC2-1) | Y | N |
| CL4041.Contig2_All | AT3G56760 | Y | N |
| Unigene16995_All | AT1G28440 | Y | N |
| Unigene7456_All | AT1G12460 | Y | N |
| Unigene12466_All | AT4G30960 (SIP3) | Y | Y |
| CL6804.Contig1_All | AT3G23000 (CIPK7) | Y | Y |
| Unigene15000_All | AT1G27190 | Y | Y |
| Unigene10496_All | AT1G71810 | N | Y |
| CL3707.Contig1_All | AT3G09010 | N | Y |
| CL12895.Contig3_All | AT3G10540 | N | Y |
| Unigene5058_All | AT3G14460 | N | Y |
| Unigene19774_All | AT4G18950 | N | Y |
| CL12723.Contig1_All | AT5G24430 | N | Y |
| CL9230.Contig1_All | AT5G63930 | N | Y |
| Unigene7537_All | AT4G33430 (BAK1) | N | Y |
| CL2576.Contig1_All | AT4G26610 (D6PKL1) | N | Y |
| Unigene53797_All | AT5G47750 (D6PKL2) | N | Y |
| Unigene4307_All | AT1G23260 (MMZ1 ) | N | Y |
| CL4023.Contig2_All | AT3G12200 (Nek7) | N | Y |
| **Transcription Factor** | | | |
| CL5207.Contig1_All | AT5G08790 (ATAF2) | Y | Y |
| CL6049.Contig3_All | AT4G05100 (MYB74) | Y | N |
| CL9206.Contig4_All | AT4G17570 (GATA26) | Y | Y |
| Unigene17539_All | AT4G36620 (GATA19) | Y | Y |
| Unigene22616_All | AT5G21960 | Y | Y |
| CL8432.Contig3_All | AT3G23220 (ESE1) | Y | Y |
| CL9880.Contig2_All | AT4G23550 (WRKY29) | Y | Y |
| Unigene10086_All | AT5G65640 (bHLH093) | Y | Y |
| CL169.Contig1_All | AT1G33060 (NAC014) | Y | Y |
| Unigene24839_All | AT4G18880 (HSFA4A) | Y | Y |
| Unigene2986_All | AT5G03720 (HSFA3 ) | Y | N |
| Unigene2778_All | AT5G16820 (HSF3) | Y | Y |
| CL74.Contig1_All | AT5G05610 (AL1) | Y | Y |
| CL6399.Contig1_All | AT3G10500 (NAC053) | Y | N |
| Unigene14772_All | AT3G58710 (WRKY69) | Y | Y |
| CL6477.Contig2_All | AT2G41130 | Y | Y |
| CL7054.Contig2_All | AT1G25560 (TEM1) | Y | Y |
| Unigene19765_All | AT3G19860 (bHLH121) | Y | Y |
| CL163.Contig5_All | AT5G20730 (NPH4) | Y | Y |
| CL1795.Contig2_All | AT4G02590 (UNE12) | Y | Y |
| Unigene1938_All | AT3G17100 | Y | Y |
| CL836.Contig1_All | AT4G34680 (GATA3) | Y | Y |
| Unigene2285_All | AT3G62420 (BZIP53) | Y | Y |
| Unigene21017_All | AT3G23240 (ERF1) | Y | Y |
| Unigene39056_All | AT1G27740 (RSL4) | Y | N |
| Unigene17244_All | AT1G68810 | Y | Y |
| CL13154.Contig2_Al | AT1G26945 (KDR) | Y | Y |
| Unigene35456_All | AT3G18400 (NAC058) | Y | N |
| Unigene2222_All | AT4G01250 (WRKY22) | Y | Y |
| CL1521.Contig1_All | AT1G52890 (NAC019) | Y | Y |
| Unigene17539_All | AT4G36620 (GATA12) | Y | Y |
| CL6936.Contig3_All | AT2G40950 (BZIP17) | Y | Y |
| CL3317.Contig1_All | AT5G47230 (ERF5) | Y | Y |
| Unigene18565_All | AT1G56010 (NAC1) | Y | Y |
| Unigene8038_All | AT5G13180 (NAC083) | Y | Y |
| CL2813.Contig2_All | AT5G57620 (MYB36) | Y | Y |
| Unigene9568_All | AT1G22640 (MYB3) | Y | Y |
| Unigene57160_All | AT5G14340 (MYB40) | Y | Y |
| Unigene7348_All | AT1G51600 (ZML2 ) | Y | N |
| Unigene20999_All | AT3G06590 | N | Y |
| Unigene12355_All | AT3G18060 | N | Y |
| CL12498.Contig2_All | AT3G23880 | N | Y |
| CL5492.Contig2_All | AT5G62300 | Y | Y |
| Unigene17075_All | AT3G50650 | N | Y |
| Unigene8179_All | AT1G69010 (BIM2) | N | Y |
| Unigene2651_All | AT1G45249 (ABF2) | Y | Y |
| **UPS Enzymes** | | | |
| Unigene21179_All | AT5G20910 (AIP2) | Y | Y |
| Unigene18607_All | AT1G10940 (ASK1) | N | Y |
| Unigene9538_All | AT1G15670 | Y | Y |
| Unigene17402_All | AT1G80440 | Y | Y |
| Unigene20253_All | AT3G09770 | Y | Y |
| Unigene1938_All | AT3G17100 | Y | Y |
| CL9874.Contig2_All | AT4G04940 | Y | Y |
| Unigene3281_All | AT4G15420 | N | Y |
| CL13327.Contig5_All | AT4G15470 | Y | Y |
| Unigene24229_All | AT5G01720 | Y | Y |
| Unigene21949_All | AT5G15550 | Y | Y |
| CL9265.Contig1_All | AT5G66240 | N | Y |
| Unigene7612_All | AT1G27840 | N | Y |
| CL11499.Contig3_All | AT2G42160 (BRIZ1) | Y | Y |
| Unigene4949_All | AT5G64920 (CIP8) | Y | Y |
| Unigene23605_All | AT5G64660 (CMPG2) | Y | Y |
| Unigene22622_All | AT1G26830 (CUL3) | Y | Y |
| Unigene2206_All | AT4G31160 (DCAF1) | N | Y |
| Unigene7288_All | AT1G76260 (DWA2) | Y | Y |
| Unigene47112_All | AT5G05560 (EMB2771) | N | Y |
| CL30.Contig3_All | AT3G54650 (FBL17) | N | Y |
| CL10027.Contig4_All | AT2G19520 (FVE) | Y | Y |
| Unigene17067_All | AT5G13480 (FY) | Y | Y |
| Unigene16959_Al | AT3G59410 (GCN2) | Y | Y |
| Unigene10187_All | AT1G02860 (NLA) | Y | Y |
| CL11138.Contig1_All | AT3G51260 (PAD1) | Y | Y |
| Unigene1863_All | AT5G40580 (PBB2) | N | Y |
| Unigene19803_All | AT2G33770 (PHO2) | Y | Y |
| Unigene14564_All | AT5G02310 (PRT6) | Y | Y |
| Unigene20994_All | AT3G46510 (PUB13) | Y | Y |
| Unigene15432_All | AT1G29340 (PUB17) | N | Y |
| Unigene20641_All | AT3G52450 (PUB22) | N | Y |
| Unigene14520_All | AT2G35930 (PUB23) | N | Y |
| CL5577.Contig2_All | AT1G48780 (PUB26) | Y | Y |
| CL2449.Contig1_Al | AT3G07360 (PUB9) | Y | Y |
| Unigene4843_All | AT5G14420 (RGLG2) | Y | Y |
| Unigene17354_All | AT4G14220 (RHF1A) | Y | Y |
| CL10365.Contig3_All | AT5G22000 (RHF2A) | Y | Y |
| CL1594.Contig1_All | AT5G51450 (RIN3) | Y | Y |
| Unigene6695_All | AT5G10380 (RING1) | Y | Y |
| Unigene10089_All | AT3G12630 (SAP5) | Y | Y |
| Unigene18868_All | AT3G57870 (SCE1 ) | Y | Y |
| Unigene21619_All | AT3G58040 (SINAT2) | Y | Y |
| CL11725.Contig1_All | AT3G47990 (SIS3) | Y | Y |
| Unigene23575_All | AT5G57900 (SKIP1) | Y | Y |
| CL7719.Contig2_All | AT1G06110 (SKIP16) | Y | Y |
| Unigene14803_All | AT3G61350 (SKIP4) | N | Y |
| Unigene11647_All | AT175950 (SKP1) | N | Y |
| Unigene19726_All | AT4G24210 (SLY1) | Y | Y |
| CL8763.Contig3_All | AT1G02840 (SR34) | N | Y |
| Unigene5528_All | AT3G62980 (TIR1) | Y | Y |
| Unigene5404_All | AT1G14400 (UBC1) | N | Y |
| Unigene5077_All | AT5G53300 (UBC10) | Y | Y |
| Unigene445_All | AT3G46460 (UBC13) | Y | Y |
| Unigene23721_All | AT4G36410 (UBC17) | Y | Y |
| Unigene603_All | AT1G50490 (UBC20) | N | Y |
| CL9822.Contig2_All | AT5G05080 (UBC22) | N | Y |
| Unigene57943_All | AT5G41340 (UBC4) | Y | Y |
| Unigene18677_All | AT1G63800 (UBC5) | N | Y |
| Unigene6798_All | AT5G59300 (UBC7) | Y | Y |
| Unigene30887_All | AT5G57990 (UBP23) | Y | Y |
| Unigene30837_All | AT1G51710 (UBP6) | Y | Y |
| Unigene18596_All | AT3G52560 (UEV1D-4) | N | Y |
| CL12517.Contig3_All | AT1G55860 (UPL1 ) | N | Y |
| CL13222.Contig1_All | AT3G17205 (UPL6 ) | Y | Y |
| Unigene37381_All | AT5G63860 (UVR8) | Y | Y |
| Unigene14425_All | AT1G47056 (VFB1 ) | Y | Y |
| Unigene18607_All | AT5G42190 (ASK2) | Y | N |
| Unigene22650_All | AT1G24440 | Y | N |
| Unigene10163_All | AT1G26930 | Y | N |
| CL3743.Contig2_All | AT2G17020 | Y | Y |
| CL1898.Contig1_All | AT2G32730 | Y | N |
| Unigene17402_All | AT2G44130 | Y | N |
| CL2038.Contig1_All | AT2G25490 (EBF1) | Y | Y |

**Table S8.** Primers used in current study.

| **Primer name** | **Sequences** |
| --- | --- |
| **qPCR primers** | |
| CL4246-QF | TCGTGGTAGAAGTGCACCAG |
| CL4246-QR | GAGTTTGGAGGCGCAGTAGA |
| Unigene22003-QF | ACATTATCCCAGCTCGCCTC |
| Unigene22003-QR | GGACAAAGCCTGCATGAAC |
| Unigene23616-QF | TCGTGGTAGAAGTGCACCAG |
| Unigene23616-QR | GAGTTTGGAGGCGCAGTAGA |
| Unigene22615-QF | CGACGGGTGAAATCTCGTTC |
| Unigene22615-QR | CTTGTCTTGCAACACTCGCT |
| Unigene61098-QF | AGTGGTGGGCAAGGTTGTAG |
| Unigene61098-QR | GTCCGATTAGTGGGACGATG |
| CL8091-QF | GGACCCCGTGGTTACATTCT |
| CL8091-QR | CTGCTCCCGATCTTCTTCCTC |
| Unigene5528-QF | TACGGTCCAAACCCCTGAAC |
| Unigene5528-QR | TGCTCTGCAAGGACCTCAAG |
| CL5577-QF | CATAGCTTCCAACCCCGAC |
| CL5577-QF | TCAAAGCAGCCGAATTGAC |
| Unigene5077-QF | CTCAGGTGCATCGATCTGGT |
| Unigene5077-QR | GATGCCATCTCGAAACCAAC |
| Unigene19871-QF | CGCTAATTCGTCACGCAGTC |
| Unigene19871-QR | GCGGCGAAGATAGGAGAAG |
| Unigene16368-QF | TTGCCTCCTGGATTTCGTTTC |
| Unigene16368-QR | AGGCAAAGGGTTGGAAAATGC |
| CL2554-QF | GTCTGAACCATGGGACTTGC |
| CL2554-QR | GCCCTATTTGTCCTCATTCCAG |
| CL6534-QF | ATGACCCTTGGGAACTTCCTG |
| CL6534-QR | AGTAGTAACTCGGTTCGGTCG |
| Unigene17402-QF | GTTTGTCCTAGAGGGCGTG |
| Unigene17402-QR | CTCCGACCGACAAACAAAG |
| Unigene20426-QF | TCTGGCTTGGAACCTTTGAC |
| Unigene20426-QR | GTTAGCACCCATGAGCTTGAG |
| Unigene 57160-QF | CGGCATGTTCCTATGCTTGC |
| Unigene 57160-QR | TCGGCTCTCTTCCATATCTGT |
| Unigene 14772-QF | CCCAGCCAGGAAACAAGTAG |
| Unigene 14772-QR | TGGTGGTGCTTTGTTGTTGG |
| Unigene 9789-QF | AGCCTCCTTTGTGTTCTGCT |
| Unigene10187-QF | CACCATGGCTATGCGAACTC |
| Unigene10187-QR | GAGGGAGCAACCCTCAAAC |
| Unigene 9789-QR | TGACAACGGCTGGAAGATGA |
| CL9880-QF | TGCAGTAGCTCAAAAGGGTG |
| CL 9880-QR | TTGGCTTAGCATGGTTGTGC |
| ZxActin-QF | TTTTCCAGCCATCCCTTGTT |
| ZxActin-QR | TGCAGTGATCTCCTTGCTCATAC |
| AtActin2-QF | Tcagatgcccagaagtgttgttcc |
| AtActin2-QR | Ccgtacagatccttcctgatatcc |
| **Full length CDS sequence primers** | |
| Unigene16368-Full-F | AAAAAGCAGGCTTCATGCAGAAAAATACTTCTTTG |
| Unigene16368-Full-R | AGAAAGCTGGGTTTGGTCTTTTTGAAAGGCAAGGA |
| CL6534-Full-F | AAAAAGCAGGCTTCATGAGCCAAGAAGATACCACC |
| CL6534-Full-R | AGAAAGCTGGGTTTTTGAAAGAACTTCTAACATCTTTG |

**References**

1. Tian W, Hou C, Ren Z, Pan Y, Jia J, Zhang H, et al. A molecular pathway for CO₂ response in Arabidopsis guard cells. Nature Commun. 2015;doi: 10.1038/ncomms7057.
2. Stenvik GE, Tandstad NM, Guo Y, Shi CL, Kristiansen W, Holmgren A, et al. The EPIP peptide of INFLORESCENCE DEFICIENT IN ABSCISSION is sufficient to induce abscission in Arabidopsis through the receptor-like kinases HAESA and HAESA-LIKE2. Plant Cell. 2008;20:1805-17.
3. Teige M, Scheikl E, Eulgem T, Dóczi R, Ichimura K, Shinozaki K, et al. The MKK2 pathway mediates cold and salt stress signaling in Arabidopsis. Mol Cell. 2004;15:141-52.
4. [Chaves-Sanjuan A](https://www.ncbi.nlm.nih.gov/pubmed/?term=Chaves-Sanjuan%20A%5BAuthor%5D&cauthor=true&cauthor_uid=25288725), [Sanchez-Barrena MJ](https://www.ncbi.nlm.nih.gov/pubmed/?term=Sanchez-Barrena%20MJ%5BAuthor%5D&cauthor=true&cauthor_uid=25288725), [Gonzalez-Rubio JM](https://www.ncbi.nlm.nih.gov/pubmed/?term=Gonzalez-Rubio%20JM%5BAuthor%5D&cauthor=true&cauthor_uid=25288725), [Moreno M](https://www.ncbi.nlm.nih.gov/pubmed/?term=Moreno%20M%5BAuthor%5D&cauthor=true&cauthor_uid=25288725), [Ragel P](https://www.ncbi.nlm.nih.gov/pubmed/?term=Ragel%20P%5BAuthor%5D&cauthor=true&cauthor_uid=25288725), [Jimenez M](https://www.ncbi.nlm.nih.gov/pubmed/?term=Jimenez%20M%5BAuthor%5D&cauthor=true&cauthor_uid=25288725), et al. Structural basis of the regulatory mechanism of the plant CIPK family of protein kinases controlling ion homeostasis and abiotic stress. Proc Nati Acad Sci U S A. 2006;111: E4532-41.
5. Amarasinghe S, Watson-Haigh NS, Gilliham M, Roy S, Baumann U. The evolutionary origin of CIPK16: A gene involved in enhanced salt tolerance. Mol Phylogenet Evol. 2016;100: 135-47.
6. Wang P, Yang Q, Sang S, Chen Y, Zhong Y, Wei Z. Arabidopsis inositol polyphosphate kinase AtIpk2β is phosphorylated by CPK4 and positively modulates ABA signaling. Biochem Biophys Res Commun. 2017;490:441-6.
7. [Ronzier E](https://www.ncbi.nlm.nih.gov/pubmed/?term=Ronzier%20E%5BAuthor%5D&cauthor=true&cauthor_uid=25037208), [Corratgé-Faillie C](https://www.ncbi.nlm.nih.gov/pubmed/?term=Corratg%C3%A9-Faillie%20C%5BAuthor%5D&cauthor=true&cauthor_uid=25037208), [Sanchez F](https://www.ncbi.nlm.nih.gov/pubmed/?term=Sanchez%20F%5BAuthor%5D&cauthor=true&cauthor_uid=25037208), [Prado K](https://www.ncbi.nlm.nih.gov/pubmed/?term=Prado%20K%5BAuthor%5D&cauthor=true&cauthor_uid=25037208), [Brière C](https://www.ncbi.nlm.nih.gov/pubmed/?term=Bri%C3%A8re%20C%5BAuthor%5D&cauthor=true&cauthor_uid=25037208), [Leonhardt N](https://www.ncbi.nlm.nih.gov/pubmed/?term=Leonhardt%20N%5BAuthor%5D&cauthor=true&cauthor_uid=25037208), et al. CPK13, a noncanonical Ca2+-dependent protein kinase, specifically inhibits KAT2 and KAT1 shaker K+ channels and reduces stomatal opening. Plant Physiol. 2014;166:314-26.
8. Guan Q, Yue X, Zeng H, Zhu J. The protein phosphatase RCF2 and its interacting partner NAC019 are critical for heat stress-responsive gene regulation and thermotolerance in Arabidopsis. Plant Cell. 2014;26:438-53.
9. Takasaki H, Maruyama K, Takahashi F, Fujita M, Yoshida T, Nakashima K, et al. SNAC-As, stress-responsive NAC transcription factors, mediate ABA-inducible leaf senescence. Plant J. 2015;84:1114-23.
10. Ding Y, Liu N, Virlouvet L, Riethoven JJ, Fromm M, Avramova Z. Four distinct types of dehydration stress memory genes in *Arabidopsis thaliana*. BMC Plant Biol. 2013;doi: 10.1186/1471-2229-13-229.
11. [Liu H](https://www.ncbi.nlm.nih.gov/pubmed/?term=Liu%20H%5BAuthor%5D&cauthor=true&cauthor_uid=20354906)^.^, [Yang W](https://www.ncbi.nlm.nih.gov/pubmed/?term=Yang%20W%5BAuthor%5D&cauthor=true&cauthor_uid=20354906), [Liu D](https://www.ncbi.nlm.nih.gov/pubmed/?term=Liu%20D%5BAuthor%5D&cauthor=true&cauthor_uid=20354906), [Han Y](https://www.ncbi.nlm.nih.gov/pubmed/?term=Han%20Y%5BAuthor%5D&cauthor=true&cauthor_uid=20354906), [Zhang A](https://www.ncbi.nlm.nih.gov/pubmed/?term=Zhang%20A%5BAuthor%5D&cauthor=true&cauthor_uid=20354906), [Li S](https://www.ncbi.nlm.nih.gov/pubmed/?term=Li%20S%5BAuthor%5D&cauthor=true&cauthor_uid=20354906). Ectopic expression of a grapevine transcription factor *VvWRKY11* contributes to osmotic stress tolerance in Arabidopsis. Mol Biol Rep. 2011; 38:417-27.
12. Zhang X, Garreton V, Chua NH. The AIP2 E3 ligase acts as a novel negative regulator of ABA signaling by promoting ABI3 degradation. Genes Dev. 2005;19:1532-43.
13. Cho SK, Ryu MY, Song C, Kwak JM, Kim WT. Arabidopsis PUB22 and PUB23 are homologous U-Box E3 ubiquitin ligases that play combinatory roles in response to drought stress. Plant Cell. 2008;20:1899-914.
